# Toxicological Effects of the Different Substances in Tobacco Smoke on Human Embryonic Development by a Systems Chemo-Biology Approach

**DOI:** 10.1371/journal.pone.0061743

**Published:** 2013-04-29

**Authors:** Bruno César Feltes, Joice de Faria Poloni, Daniel Luis Notari, Diego Bonatto

**Affiliations:** 1 Department of Molecular Biology and Biotechnology, Biotechnology Center of the Federal University of Rio Grande do Sul, Federal University of Rio Grande do Sul, Porto Alegre, RS – Brazil; 2 Institute of Biotechnology, University of Caxias do Sul, Caxias do Sul, RS – Brazil; 3 Computational and Information Technology Center, Universidade de Caxias do Sul, Caxias do Sul, RS – Brazil; Laboratoire de Biologie du Développement de Villefranche-sur-Mer, France

## Abstract

The physiological and molecular effects of tobacco smoke in adult humans and the development of cancer have been well described. In contrast, how tobacco smoke affects embryonic development remains poorly understood. Morphological studies of the fetuses of smoking pregnant women have shown various physical deformities induced by constant fetal exposure to tobacco components, especially nicotine. In addition, nicotine exposure decreases fetal body weight and bone/cartilage growth in addition to decreasing cranial diameter and tibia length. Unfortunately, the molecular pathways leading to these morphological anomalies are not completely understood. In this study, we applied interactome data mining tools and small compound interaction networks to elucidate possible molecular pathways associated with the effects of tobacco smoke components during embryonic development in pregnant female smokers. Our analysis showed a relationship between nicotine and 50 additional harmful substances involved in a variety of biological process that can cause abnormal proliferation, impaired cell differentiation, and increased oxidative stress. We also describe how nicotine can negatively affect retinoic acid signaling and cell differentiation through inhibition of retinoic acid receptors. In addition, nicotine causes a stress reaction and/or a pro-inflammatory response that inhibits the agonistic action of retinoic acid. Moreover, we show that the effect of cigarette smoke on the developing fetus could represent systemic and aggressive impacts in the short term, causing malformations during certain stages of development. Our work provides the first approach describing how different tobacco constituents affect a broad range of biological process in human embryonic development.

## Introduction

There are more than 4,800 compounds present in the particulate and vapor phases of cigarette smoke [1], and many of these compounds are considered to represent a human health risk [2]. Known constituents of cigarette smoke include isoprene, butadiene, polycyclic aromatic hydrocarbons (PAHs), aldehydes, metals, *N*-nitrosamines, and aromatic amines, in addition to many others [1]. Although extensive anti-tobacco public advertisements promote smoking cessation in pregnant women, a considerable number of women still smoke during their pregnancies and/or are exposed to tobacco smoke via passive smoking [2], [3], [4].

We addressed two major issues in this work. Although prenatal smoke exposure has been previously associated with innumerable malformations during fetus growth and development and disruptions of reproductive physiology, there are gaps in the knowledge of how tobacco components (TCs) affect the developing embryo in pregnant women in a systemic way, [2], [3], [5], [6]. This knowledge gap is the first issue that we address. Interestingly, these abnormalities are not tissue specific or related to any unique pathway but, rather, are systemic and connected to a broad range of birth defects [2], [4]. The second issue that we address relates to the fact that nicotine is the principal psychoactive constituent of tobacco, understanding its biological effects on fetal and maternal health is critical, as it may affect distinct biochemical pathways when compared to other tobacco smoke constituents. Studies concerning the morphological effects of tobacco smoke constituents in fetuses from both active and passive smoking women have shown significant alterations in weight, fat mass and most anthropometric parameters as well as in the placenta with alterations in protein metabolism and enzyme activity [7]. These alterations are the results of a direct toxic effect on the fetal cells or an indirect effect through damage to, and/or functional disturbances of the placenta [7]. One possible explanation that could link nicotine and the negative regulation of development is retinoic acid (RA) signaling. RA is an indispensable molecule involved in the regulation of gene expression and cell-cell signaling during early development [8]. RA can cross the cell membrane and bind to specific nuclear receptors, such as retinoic acid receptors (RARs) and retinoid × receptors (RXRs) [8]. Studies regarding the role of RA receptors during embryogenesis have shown that RARs are essential for the expression of HOX genes and skeletal development [8], [9]. Nicotine has been previously associated with inhibition of the RARβ gene in lung cancer, which suggests that nicotine affects RA signaling in human tissues [10]. Therefore, RA signaling is a plausible pathway through which nicotine could affect cell differentiation and cause human fetal morphological abnormalities. However, the molecular mechanisms underlying the progression or the cause of fetal abnormalities related to cigarette smoking remain unknown.

To understand these mechanisms, we performed systems chemo-biology analyses to elucidate the nature and number of proteins and modules that are associated with prenatal tobacco smoke exposure. Different protein-protein interaction (PPI) and chemical-protein interaction (CPI) networks derived from interactome projects were described. In a first analysis, we prospected and analyzed a network using a list of 95 commonly found harmful tobacco constituents [2], to elucidate how these substances could act together to influence embryonic and fetal development. In a second systems chemo-biology analysis, we prospected data on the interactome and small compounds for nicotine alone and examined how they could negatively affect cell differentiation and bone development and lead to morphological abnormalities. Furthermore, we conducted gene ontology (GO) analyses of the major biological processes derived from the PPI and CPI networks. Supporting the hypotheses gathered from systems chemo-biology analyses, a landscape network study was performed using available transcriptomic data of placenta and cord blood isolated from passive smoking women and non-smoking women [11].

A model of how selected TCs could influence embryonic development was generated. We also developed a separate model of how nicotine could affect cell differentiation and bone development. Taken together, our systems chemo-biology data are the first to show how tobacco smoke can affect fetal and embryonic development in a systemic matter at the molecular level.

## Materials and Methods

### Interactome Data Mining and Design of the Chemo-biology Network

To design chemo-biology interactome networks and to elucidate the interplay between development and TCs, the metasearch engines STITCH 3.1 [http://stitch.embl.de/] and STRING 9.0 [http://string-db.org/] [12], [13] were used. In this sense, a list of 51 commonly found TCs, many of them with known concentrations in the mainstream and sidestream tobacco smoke [2] were used as initial seed for network prospection in STITCH. STITCH software allows visualization of the physical connections among different proteins and chemical compounds, whereas STRING shows protein-protein interactions. Each protein-protein or protein-chemical connection (edge) shows a degree of confidence between 0 and 1.0 (with 1.0 indicating the highest confidence). The parameters used in STITCH software were as follows: all prediction methods enabled, excluding text mining; 20 to 50 interactions; degree of confidence, medium (0.400); and a network depth equal to 1. The results gathered using these search engines were analyzed with Cytoscape 2.8.2 [14]. In addition, the GeneCards [http://www.genecards.org/] [15], [16], KEGG [http://www.genome.jp/kegg/] [17], iHop [http://www.ihop-net.org/UniPub/iHOP/] [18], PubChem [http://pubchem.ncbi.nlm.nih.gov/], ALOGPS 2.1 [http://www.vcclab.org/lab/alogps/] [19], AmiGO 1.8 [http://amigo.geneontology.org/cgi-bin/amigo/go.cgi] [20], and Gene Expression Atlas [http://www.ebi.ac.uk/gxa/] [21] search engines were also employed using their default parameters.

To prospect protein-protein and chemical-protein interactions (PPI and CPI, respectively), we entered each TC into the STITCH program. TCs that were not present in the STITCH database (or those that did not shown any protein connections) and particularly well described components, such as nitric oxide, phenol and carbon monoxide, were excluded from the analysis.

Different small CPI and PPI networks were obtained (data not shown), and these networks were further analyzed using Cytoscape 2.8.2. Each network generated by STITCH and STRING was combined into a large network using the Advanced Merge Network function, which was fully implemented in Cytoscape software.

### Gene Expression Data for the Main Associated Nodes of Tobacco Components

**Figure 1:**
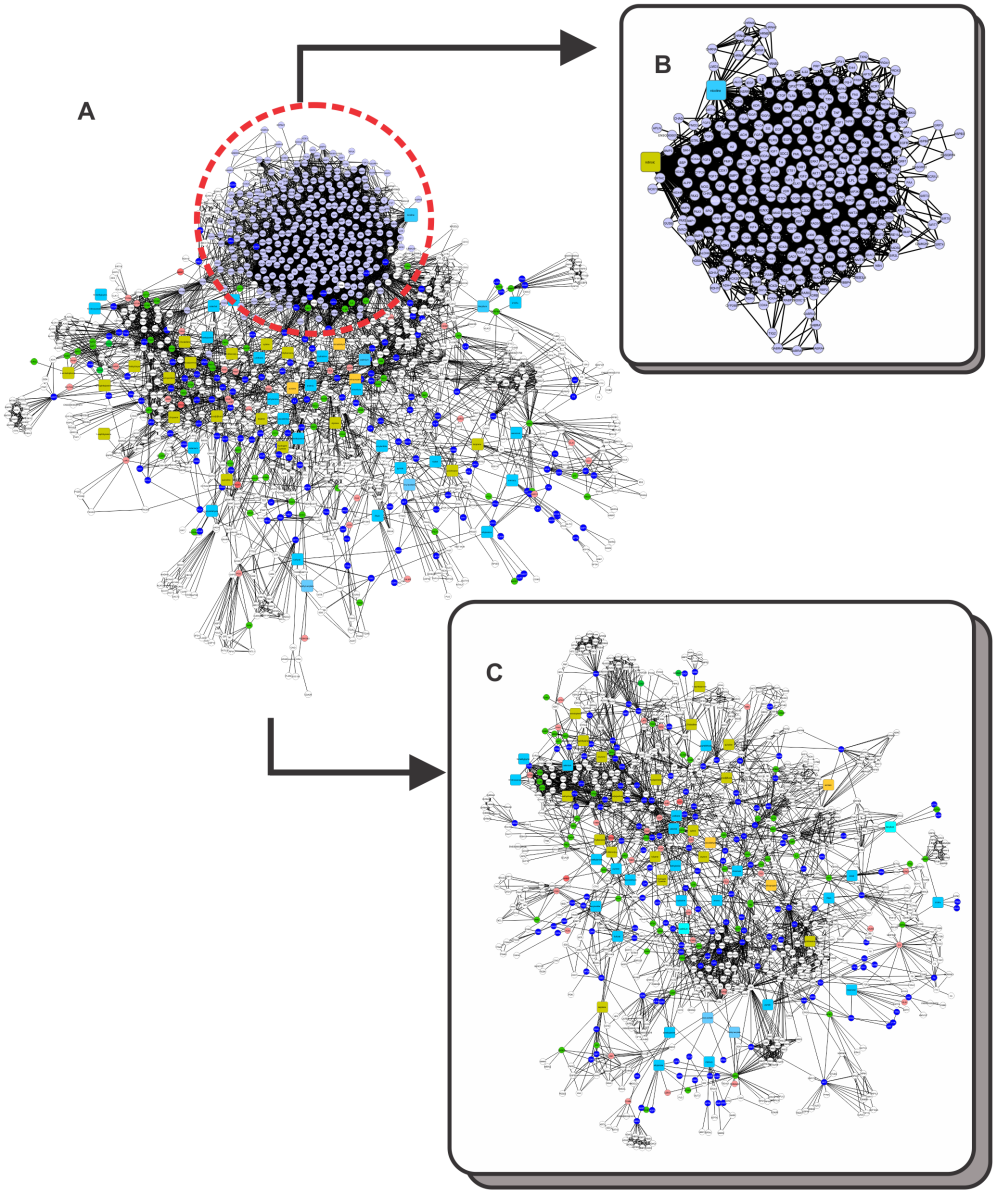
A binary network of chemical-protein and protein-protein interactions (CPI-PPI network) generated by the program Cytoscape 2.8.2. (A) The main network, showing 49 known substances present in tobacco, 1177 nodes (49 substances, 1128 proteins) and 7522 edges (connections). Proteins were colored to identify the tissue in which they were present: (i) pink indicates fetal tissue; (ii) green, embryonic tissue; and (iii) dark blue, both fetal and embryonic tissues. In addition, each substance was colored according to its solubility: (i) yellow indicates lipophilic and (ii) light blue, hydrophilic. We observed that nicotine resided in a module apart from the major network (A). Therefore, we separated it from the major CPI-PPI network and colored its module purple. (B) The nicotine subnetwork is shown separately from the major CPI-PPI network. It contained proteins related to retinoic acid signaling and retinoic acid (lipophilic molecule). (C) The final major CPI-PPI network after the nicotine module was extracted.

To determine whether mRNA sequences associated with specific proteins connected to each TC could be present during development, we searched the transcriptome data from the Gene Expression Atlas [22]. We used the protein name and expression data for *Homo sapiens* embryos and fetuses as the initial inputs. The expression data indicated overexpressed and underexpressed genes (Table S1 in Supporting Information S1). Gene Expression Atlas infers the expression data for a specified gene by providing a list of experimental studies [22]. We considered a gene overexpressed or underexpressed based on the number of studies that matched the expression state of our input. Proteins that are only present in embryonic tissue were colored green, whereas proteins that are only present in fetal tissue were colored pink (Table S1 in Supporting Information S1). The blue nodes indicate the presence of a protein in both embryonic and fetal tissue (Table S1 in Supporting Information S1). Uncolored nodes (default color white) connected to TCs were either not present in any of the selected tissues in the initial input or were not found in the Gene Expression Atlas database (**Fig. 1**).

Additionally, we evaluated the transcriptomic data gathered from placenta and cord blood of passive smoking women (termed group “a”), with cord blood cotinine levels >1.0 ng/mL, and from non-smoking women (group “b”), with cord blood cotinine levels <0.15 ng/mL [11]. For this purpose, the matrix file GSE30032 (available at Gene Expression Omnibus [http://www.ncbi.nlm.nih.gov/geo]) was used and a mean value of expression for each gene was generated for both groups “a” and “b”. The mean value of expression was then overlaid in CPI-PPI-derived subnetworks with the software ViaComplex 1.0 [23]. By providing gene expression data and interactomic networks, the software ViaComplex generates a landscape view of gene expression in a specific network.

### Solubility Predictions for Major Tobacco Component-associated CPI-PPI Networks

To predict the solubility of each TC in an aqueous environment, such as in blood and plasma, we used the program ALOGPS 2.1 [http://www.vcclab.org/lab/alogps/]. ALOGPS allows simulation of the probable solubility of a given compound determined based on its structural formula or CAS number. Compounds with a solubility of less than 35 g/L [values of ALOGpS and logS (exp)] were considered lipophilic. ALOGPS 2.1 was used with its default parameters.

### Module Analysis of Major Tobacco Component-associated CPI-PPI Networks

The large CPI-PPI network obtained from the initial search (**Fig. 1**) was analyzed in terms of the major cluster or module composition using the program Molecular Complex Detection (MCODE) [24], which is available at http://baderlab.org/Software/MCODE. MCODE is based on vertex weighting by the local neighborhood density and outward traversal from a locally dense seed protein to isolate the dense regions according to given parameters stipulated by the researcher [24]. The parameters for cluster finding were as follows: loops included; degree cutoff, 2; expansion of a cluster by one neighbor shell allowed (fluff option enabled); deletion of a single connected node from clusters (haircut option enabled); node density cutoff, 0.1; node score cutoff, 0.2; kcore, 2; and maximum network depth, 100. Each cluster generates a value of “cliquishness” (C*i*), which is the degree of connection in a given group of proteins. Thus, the higher the C*i* value, the more connected the cluster [24].

### Centrality Analysis of the Major Tobacco Component-associated CPI-PPI Networks

Centrality analysis was performed using the program CentiScaPe 1.2 [25]. In this analysis, the CentiScaPe algorithm evaluates each network node according to the node degree, betweenness and closeness to establish the most “central” nodes (proteins/chemicals) within the network. Thus, the most relevant node for a determined biochemical pathway or module can be obtained and further analyzed. In general terms, the closeness analysis (1) indicates the probability that any protein/chemical compound (node in our network) is relevant to another protein/chemical compound (node) in a signaling network or its associated network [25], as determined using Equation (1):
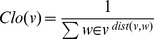
(1)where the closeness value of node *v* (*Clo(v)*) is determined by computing and totalizing the shortest paths among node *v* and all other nodes (*w*; *dist(v,w)*) found within a network (1). The average closeness (*Clo*) score was obtained by calculating the sum of different closeness scores (*Clo_i_*) divided by the total number of nodes analyzed (*N(v)*) (Equation 2).



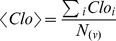
(2)The higher the closeness value compared to the average closeness score, the higher the relevance of the protein/chemical compound to other protein nodes within the network/module. In turn, the betweenness indicates the number of the shortest paths that go through each node (Equation 3) [25], [26]:
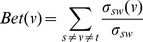
(3)where *σ_sw_* total number of the shortest paths from node *s* to node *w*, and *σ_sw_ (v)* is the number of those paths that pass through the node. The average betweenness score (*Bet*) of the network was calculated using equation (4), where the sum of different betweenness scores (*Bet_i_*) is divided by the total number of nodes analyzed (*N(v)*):*)*].



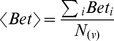
(4)Thus, nodes with high betweenness scores compared to the average betweenness score of the network are responsible for controlling the flow of information through the network topology. The higher a node’s betweenness score, the higher the probability that the node connects different modules or biological processes, such nosed are called bottleneck nodes.

Finally, the node degree (*Deg(v)*) is a measure that indicates the number of connections (*Ei*) that involve a specific node (*v*) (Equation 5):
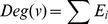
(5)


The average node degree of a network (*Deg*) is given by equation 6, where the sum of different node degree scores (*Bet_i_*) is divided by the total number of nodes (*N(v)*) present in the network:
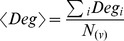
(6)


Nodes with a high node degree are called hubs [25] and have key regulatory functions in the cell.

### Gene Ontology Analyses of Major Tobacco Component-associated CPI-PPI Networks

The CPI-PPI modules generated by MCODE were further studied by focusing on major biology-associated processes using the Biological Network Gene Ontology (BiNGO) 2.44 Cytoscape plugin [27], available at http://www.cytoscape.org/plugins2.php#IO_PLUGINS. The degree of functional enrichment for a given cluster and category was quantitatively assessed (*p*-value) using a hypergeometric distribution. Multiple test correction was also assessed by applying the false discovery rate (FDR) algorithm [28], which was fully implemented in BiNGO software at a significance level of *p*<0.05. The most statistically relevant processes were taken into account when developing the interaction model.

## Results and Discussion

### Data Prospecting and Topological Design of a Major CPI-PPI Network of Different Tobacco Constituents

Systems chemo-biology tools allow interactome networks of high-throughput data to be designed for CPI and PPI networks. In this sense, systems chemo-biology and systems pharmacology tools have been employed in different research areas, like prospection of new anticancer drugs [29], in order to evaluate the interaction of different small molecules with proteins and the main biological pathways potentially affected by these compounds.

Initially, our analysis was based on a list containing 95 TCs, extracted from [2]. From this initial list, we excluded compounds such as carbon monoxide, nitric oxide and phenol, which have different pleiotropic effects within a cell and could lead to the overrepresentation of many biological pathways not directly linked to development. In addition, we excluded all compounds without any protein target described, resulting in a final list containing 51 TCs commonly found in the mainstream and sidestream tobacco smoke (Table S2 in Supporting Information S1).

We have examined the relationship between 51 TCs and embryonic development pathways using systems chemo-biology tools. It should be noted that many of the thousands of substances in tobacco smoke are considered to represent public hazards, and some have carcinogenic potential [2]. Despite the growing interest in the elucidation of molecular pathways that can be affected by these compounds, many TCs do not have a known molecular target in the cell. However, our selected list of 51 TCs represents those substances with well described concentration in tobacco smoke, making them particularly attractive for experimental hypothesis testing. Moreover, these 51 TCs have some type of interaction with proteins already described, allowing systems chemo-biology studies. From this initial list of 51 TCs, we generated 51 small CPI-PPI networks (data not shown). Both STRING and STITCH add the nodes with the highest probability to be connected to a given node. Therefore, to create different CPI-PPI networks, we identified 20 to 50 additional proteins linked to each compound using only STITCH and STRING data and merged all of the networks using the Advanced Network Merge tool, which generates a single large network (referred to as the “main network”, **Fig. 1A**). After creating the small networks, we found that RA receptors were present in the nicotine network. We decided to expand the nicotine network by adding a small network including RA and proteins related to RA signaling and embryonic development (**Fig. 1B**). The nicotine module was extracted from the first network to be studied independently because it showed a distinct module within the main network.

The resultant network after the nicotine module was extracted was referred to as the “major CPI-PPI network” and was composed of 898 nodes and 3,452 edges (**Fig. 1C**). It should be noted that, after merging each of the small CPI-PPI networks, two substances, 3-aminobiphenyl and dicyclohexyl, did not display any proteins in common with other compounds and were excluded from the analysis. Remarkably, the major CPI-PPI network did not show a wide overlap among the nodes, which indicates that TCs may have a broad influence and most likely affect different bioprocesses.

We next aimed to strengthen our understanding of our networks. We examined two types of data: (i) transcriptome data for each node directly associated with TCs to clarify whether the mRNA and, by inference, the proteins were present in the fetus (pink color), embryo (green color) or both (blue color) (Fig. 1, Table S1 in Supporting Information S1); and (ii) solubility predictions for the TCs and how this factor may influence the developing organism by characterizing each TC as hydrophilic or lipophilic (hydrophobic) (Fig. 1, Table S2 in Supporting Information S1). Nodes that did not show expression were left with uncolored (white) (Fig. 1, Table S1 in Supporting Information S1).

Interestingly, the majority of the nodes (145 of 234 total nodes; Fig. 1) have some role in human embryonic development, and thus, may affect the development of the organism. To predict TC solubility, we used the program ALOGPS 2.1. Among 48 TCs in our major CPI-PPI network (**Fig. 1**), we identified 21 lipophilic compounds and 27 hydrophilic components. Of the 27 hydrophilic components, 10 are inorganic, and 17 are organic (Table S2 in Supporting Information S1).

**Figure 2:**
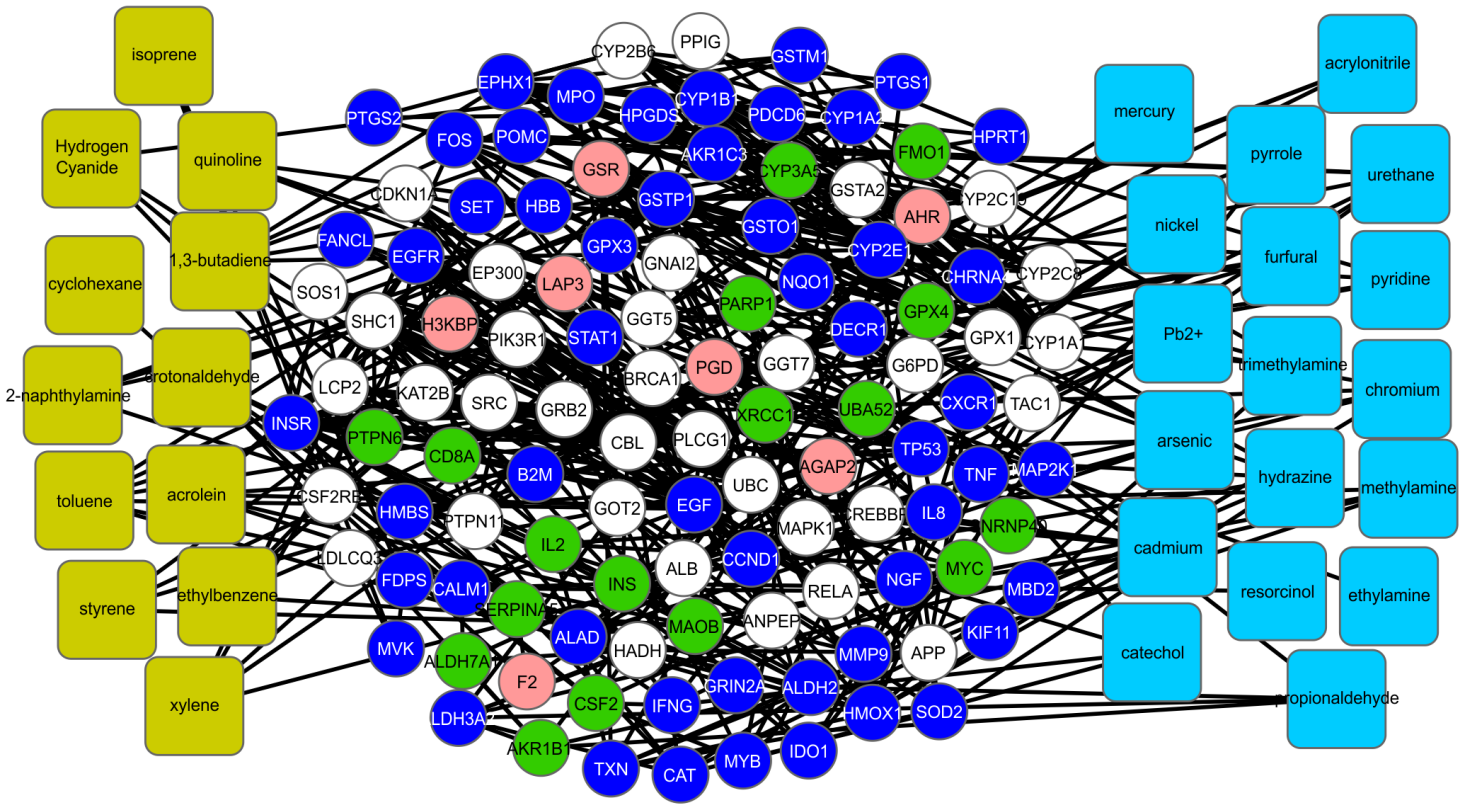
HBs found in the major CPI-PPI network. Betweenness and node degrees were assessed using the program CentiScaPe. Among the 143 HBs, 53 proteins are present in both tissues, reinforcing the idea of a prolonged effect of CS on embryonic development.

In addition, we used the program CentiScaPe 1.2 to examine the major CPI-PPI network for the most relevant proteins/compounds (**Figs. 1** and **2**). In a scale-free biological network, the most important nodes are the so called hub-bottlenecks (HBs) [30] because they combine the bottleneck function (nodes that controlling the information flow in a given network and displaying a betweenness score above the network average) and property hubs (nodes with a number of connections above the average node degree value of the network). Thus, HBs are critical nodes in a biological network [30]. In our analysis, we observed 143 HB nodes, of which 30 are TCs, and 53 were marked as present in both the fetus and embryo, 17 only in the embryo, 7 only in the fetus and 36 in neither the fetus nor embryo (white nodes) (**Fig. 2**). White nodes present in all of the networks are either not connected directly with the selected compound or do not show expression in any of the selected tissues. Because we only colored the direct nodes associated with a TC, it is clear that the TCs have a broad impact during development, acting in critical nodes that are necessary for development.

Furthermore, we sought to evaluate which TCs have the broadest effects on the major CPI-PPI network. Therefore, a closeness analysis was performed. Considering that the nodes showing the highest closeness are most relevant to the greatest number of nodes in a network [25], it can be assumed that the TCs exhibiting the highest closeness are those with the greatest systemic effects and impact the greatest number of proteins. A graph of closeness and betweenness was generated, showing that 33 TCs (from a total of 48) present closeness value above the average closeness of the network (Fig. S1 in Supporting Information S2). This finding is consistent with our interpretation that TCs have a systemic effect, impacting different proteins and physiological processes.

**Figure 3:**
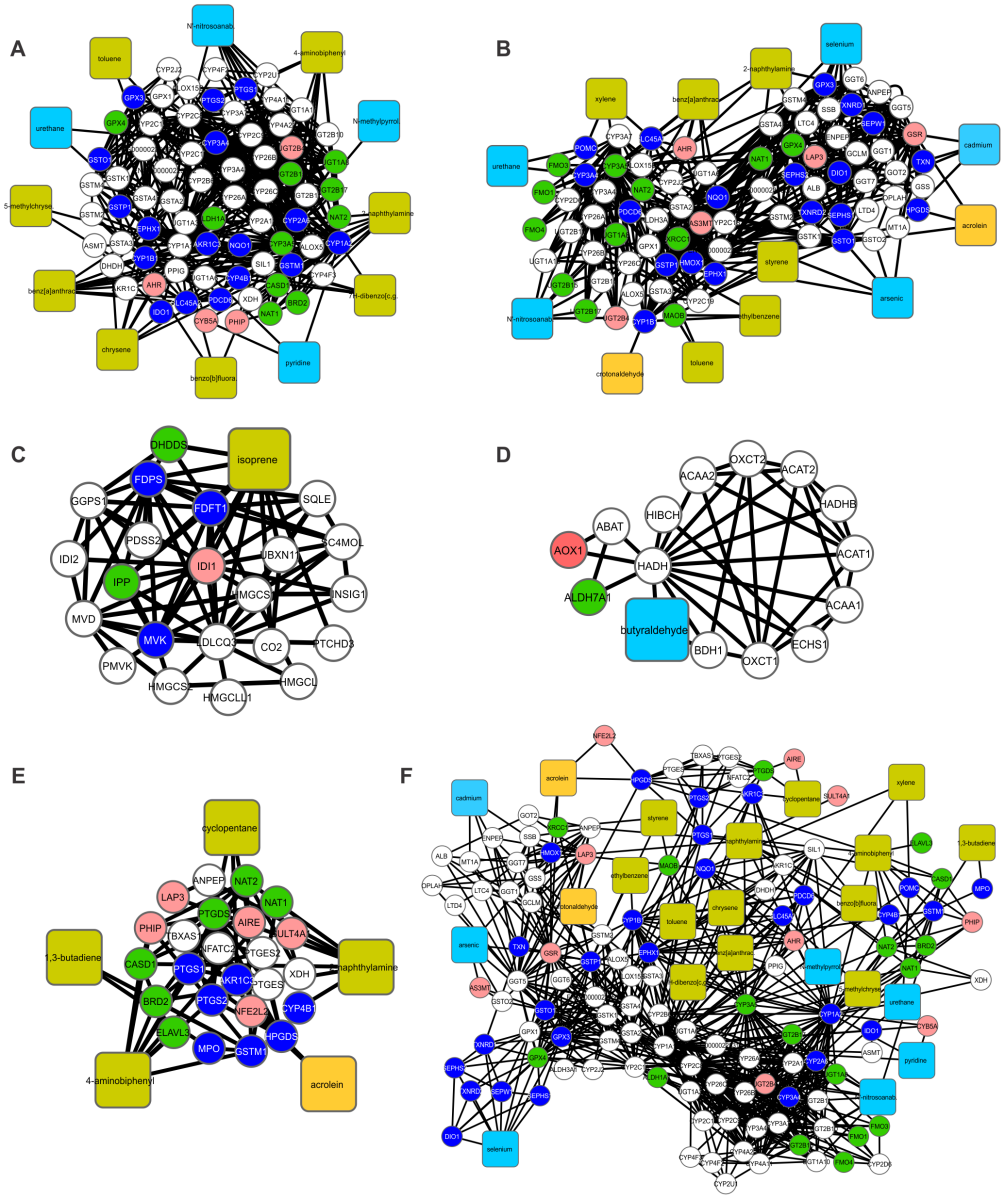
Cluster analysis of the major CPI-PPI network indicating clusters 1, 4, 11, 16 and 20. Cluster 1 (A) is composed of 83 nodes and 565 edges, with C*i* = 6,843. The associated hydrophilic constituents are urethane, *N*-nitrosoanabasine, *N*-methylpyrrolidine and pyridine. The lipophilic constituents are toluene, 4-aminobiphenyl, 5-methylcrysene, benz[a]anthracene, chrysene, benzo[b]fluoranthene, 7H-dibenzo[c,g]carbazole and 2-naphthylamine. Related GO terms: oxidation reduction and unsaturated fatty acid metabolic processes. Cluster 4 (B) is composed of 90 nodes and 411 edges, with C*i* = 4,567. The associated hydrophilic compounds are urethane, *N*-nitrosoanabasine, *N*-methylpyrrolidine, arsenic, selenium and cadmium, and the lipophilic compounds are acrolein, crotonaldehyde, toluene, xylene, ethylbenzene, benz[a]anthracene, styrene and 2-naphthylamine. Related GO term: oxidation reduction. Cluster 11 (C) is composed of 23 nodes and 74 edges, with C*i* = 3,217. Only the lipophilic compound isoprene is present in this cluster. Related GO term: steroid biosynthetic processes. Cluster 16 (D) is composed of 15 nodes and 36 edges, with C*i* = 2,400. The associated hydrophilic compound is butyraldehyde. Related GO term: lipid modification. Cluster 20 (E) is composed of 29 nodes and 65 edges, with C*i* = 2,241. The associated lipophilic compounds are acrolein, 2-naphthylamine, 1,3-butadiene, cyclopentane and 4-aminobiphenyl. Related GO terms: prostaglandin metabolic processes and unsaturated fatty acid metabolic processes. A merge of clusters 1, 4 and 20 (F). Clusters 11 and 16 did not show any proteins overlapping with any other cluster.

To understand how TCs interact with their targets, we analyzed the major CPI-PPI network for modules. From these analyses, we obtained the major TCs that affect different modules. After extraction of the nicotine subnetwork, MCODE found 22 significant modules (**Figs. 3** **–** **7**). Once the modules were obtained (**Figs. 3** **–** **7**), a gene ontology (GO) analysis was performed. Biological processes that are important for the development of organisms were listed (Table S3 in Supporting Information S1). Likewise, we performed additional GO analyses for the selected HBs (**Table 1**) and in each cluster (Tables S4–S25 in Supporting Information S1). Clusters that were not associated with significant GO terms due to a lack of data or were highly speculative in our analysis were excluded (Tables S13, S15 to S18, S22 and S25 in Supporting Information S1, Fig. S2 in Supporting Information S2).

**Figure 4:**
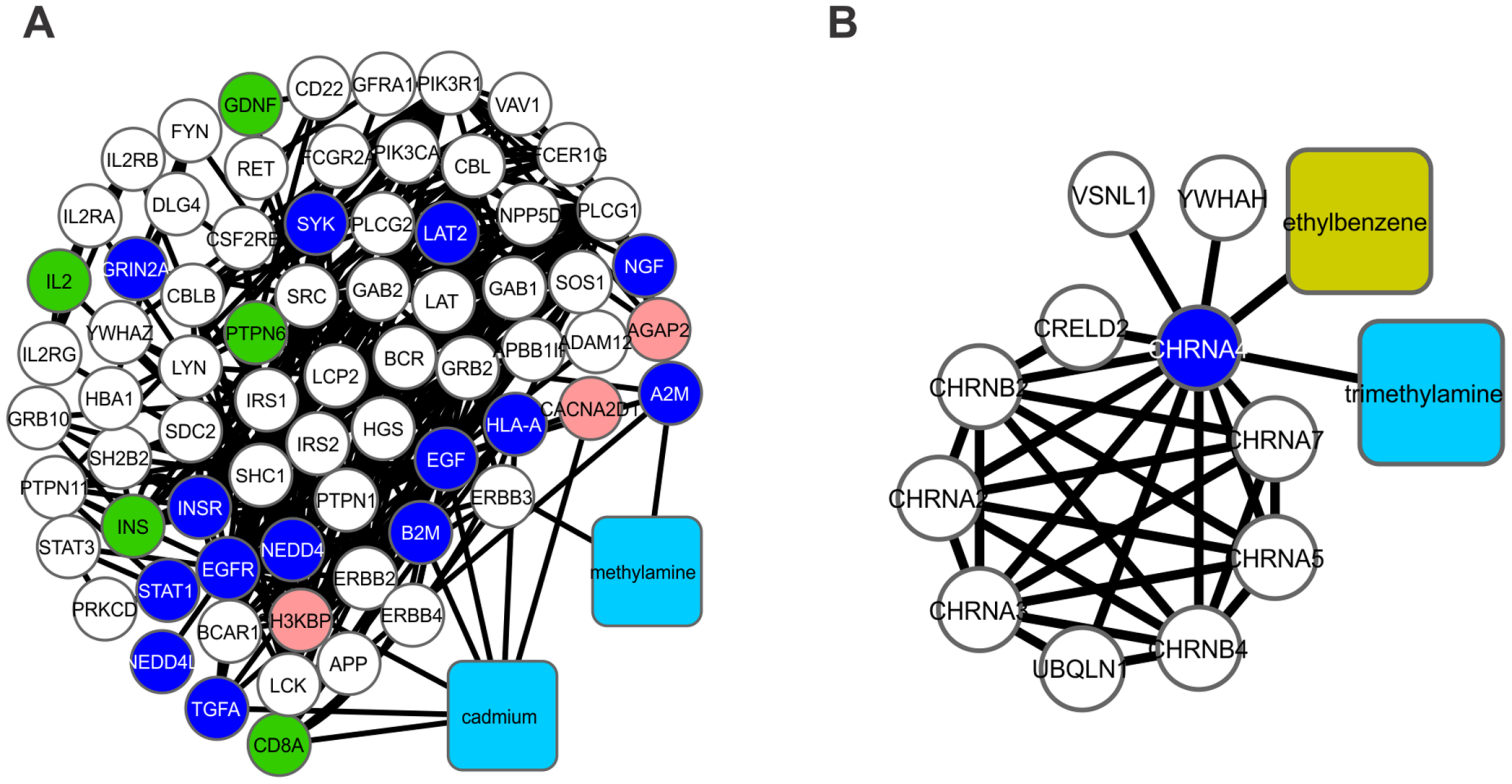
Cluster analysis of the major CPI-PPI network and the modules related to cell-cell signaling. Cluster 2 (A) is composed of 73 nodes and 354 edges, with C*i* = 4,849. Cluster 2 contains the two hydrophilic substances cadmium and methylamine. Related GO term: regulation of cell communication. Cluster 18 (B) is composed of 13 nodes and 30 edges, with C*i* = 2, 304. Cluster 18 contains one hydrophilic compound, trimethylamine, and one lipophilic compound, ethylbenzene. Related GO term: cell-cell signaling.

**Figure 5:**
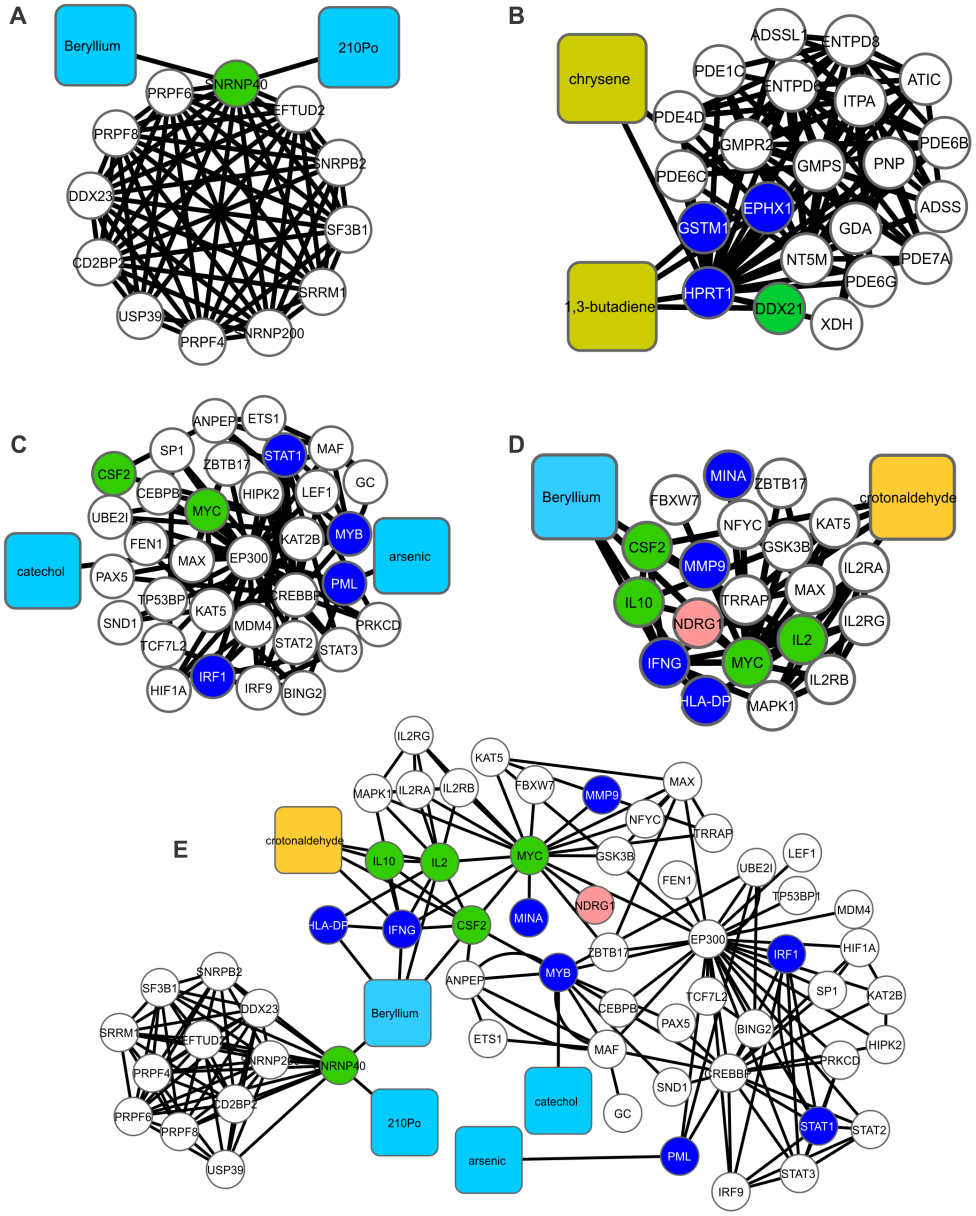
A merge of clusters 3, 6, 17 and 21. In (A), cluster 3 is composed of 14 nodes and 65 edges, with C*i* = 4,643. The associated hydrophilic components are urethane, beryllium and polonium-210. Related GO terms: RNA-splicing and nucleobase, nucleoside, nucleotide and nucleic acid metabolic processes. Cluster 6 (B) is composed of 24 nodes and 102 edges, with C*i* = 4,250. The associated lipophilic constituents are 1,3-butadiene and chrysene. Related GO term: nucleobase, nucleoside and nucleotide metabolic processes; cluster 17 (C) is composed of 35 nodes and 83 edges, with C*i* = 2,371. The hydrophilic constituents present include catechol and arsenic. Related GO term: regulation of nucleobase, nucleoside nucleotide and nucleic acid metabolic processes. Cluster 21 (D) is composed of 22 nodes and 49 edges, with C*i* = 2,227. The associated hydrophilic constituent is beryllium, and the lipophilic constituent is crotonaldehyde. Related GO term: regulation of DNA metabolic processes. The union of clusters 3, 17 and 21 (E). Cluster 6 did not show any proteins overlapping with any other cluster.

**Figure 6:**
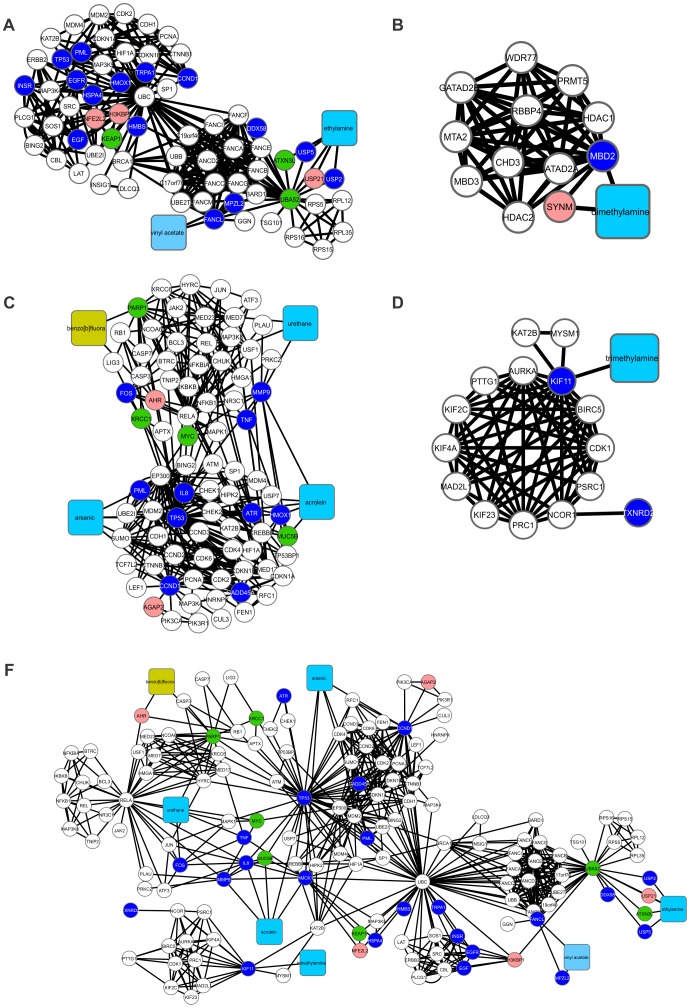
Subnetworks derived from the merge of clusters 5, 8 and 9. In (A), Cluster 5 is composed of 69 nodes and 315 edges, with C*i* = 4,565. The associated hydrophilic components are vinyl acetate and ethylamine. Related GO terms: response to DNA-damage stimulus and cell cycle. cluster 7 (B) is composed of 13 nodes and 54 edges with C*i* = 4,154. The associated hydrophilic compound is dimethylamine. Related GO term: chromatin organization. Cluster 8 (C) is composed of 85 nodes and 338 edges with C*i* = 3,976. The associated lipophilic constituents are acrolein and benzo[b]fluoranthene, whereas the hydrophilic constituents are urethane and arsenic. Related GO terms: DNA-damage stimulus and regulation of cell cycle. Cluster 9 (D) is composed of 16 nodes and 58 edges, with C*i* = 3,625. The hydrophilic constituent present is trimethylamine. Related GO term: cell cycle processes. The union of clusters 5, 8 and 9 (F). Clusters 7 did not show any proteins overlapping with any other cluster.

**Figure 7:**
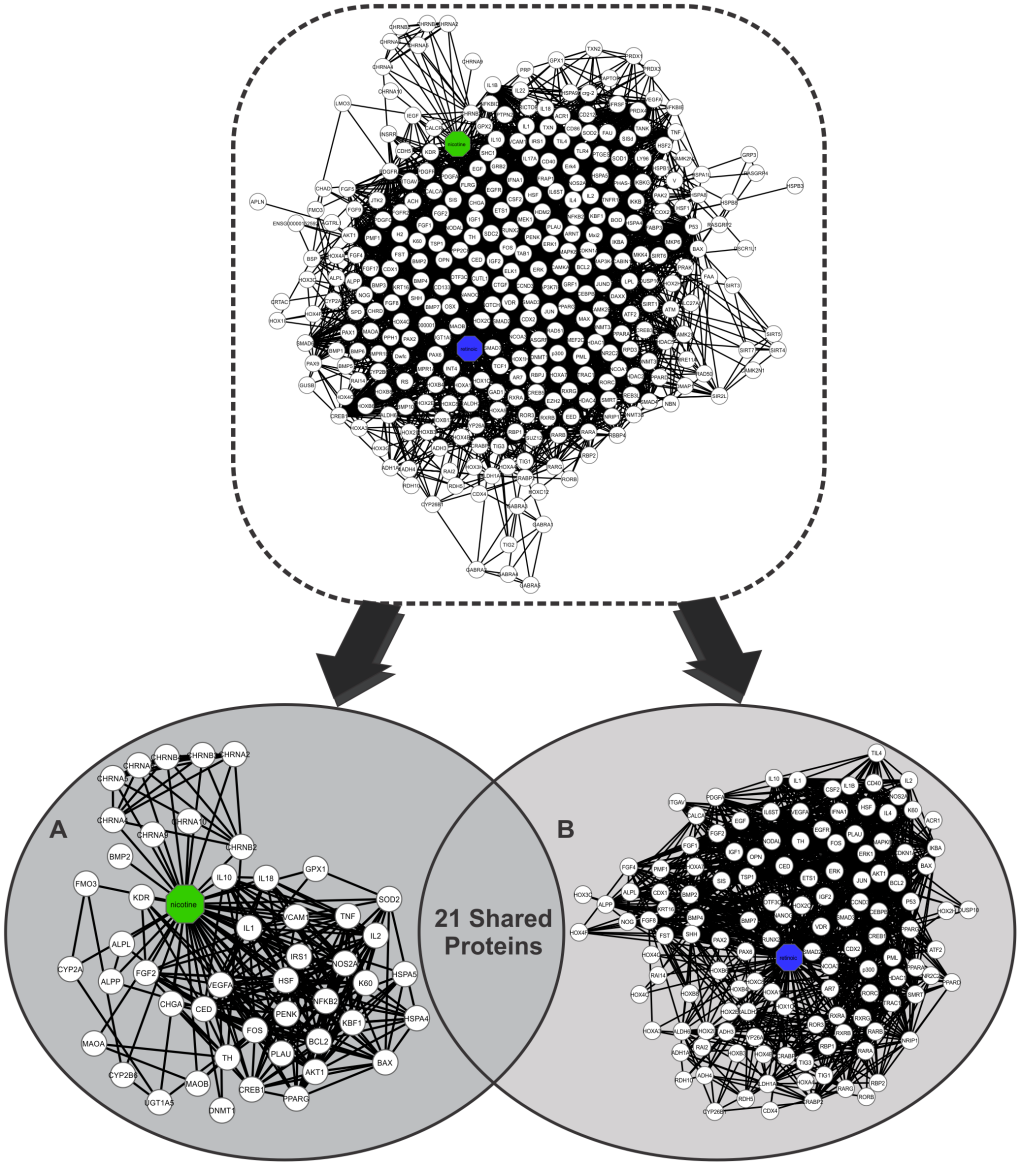
A binary network of the interactions between chemical compounds and proteins generated by the program Cytoscape 2.6.3, which contained 330 proteins and 4078 connections. Nicotine appears in the network as the green node, and RA appears as the blue node. White nodes are connected to both compounds are proteins. A) A subnetwork generated by the program Cytoscape containing 49 nodes and 281 edges and showing the proteins with direct connections with nicotine. B) A subnetwork generated by the program Cytoscape containing 130 nodes and 1,471 edges and showing the proteins that make direct connections with RA.

**Table 1:** Major bioprocesses associated with the hub-bottleneck subnetwork.

GO-ID	GO	*p*-value	Corrected *p*-value	*k* *	n#	Proteins
55114	Oxidation-reduction	4.4×10^−16^	9.0×10^−14^	26	645	CYP3A5;CYP1B1;PTGS2;CYP2C19;CYP2B6;PGD;PTGS1; ALDH3A2;AKR1C3;GSR;GPX1;FMO1;GPX4;HMOX1; GPX3;CAT;NQO1;HADH;CYP1A1;CYP2C8;MAOB;IDO1; CYP2E1;CYP1A2;DECR1;SOD2;LDLCQ3;ALDH7A1;G6PD; AKR1B1;TXN;ALDH2;MPO
48545	Regulation of steroidhormone stimuli	1.3×10^−13^	1.8×10^−11^	18	225	TNF;PTGS2;MAP2K1;RELA;PTGS1;MAOB;BRCA1; MAPK1;FOS;CCND1;CDKN1A;EP300;HMOX1;GPX4; GPX3;ALDH2;INSR;NGF
42127	Regulation of cellproliferation	1.3×10^−12^	1.5×10^−10^	30	848	CSF2;TNF;GNAI2;PTGS2;PTGS1;TAC1;GPX1;INS;HMOX1;IFNG;SHC1;EGF;INSR;MYC;EGFR;KAT2B;IL8;MAP2K1; RELA;TP53;IDO1;STAT1;MBD2;BRCA1;SOD2;MAPK1; CDKN1A;CCND1;IL2;NGF
42981	Regulation of apoptosis	9.3×10^−12^	9.4×10^−10^	29	282	CSF2;TNF;PTGS2;MMP9;GPX1;APP;INS;ALB;HMOX1; SOS1;IFNG;CAT;NQO1;EGFR;RELA;GRIN2A;TP53;IDO1; STAT1;BRCA1;SOD2;MAPK1;CDKN1A;F2;MPO;PDCD6; GSTP1;IL2;NGF
10646	Regulation of cellcommunication	6.0×10^−10^	2.6×10^−8^	31	1154	CSF2;TNF;GNAI2;PTGS2;CD8A;GRB2;TAC1;GPX1;APP; INS;SOS1;HMOX1;IFNG;CHRNA4;SHC1;CAT;EGF;INSR; AGAP2;EGFR;MAP2K1;RELA;MAOB;GRIN2A;TP53; MBD2;PTPN11;LAP3;CCND1;IL2;NGF

* Number of nodes for a given GO in the network;

# Total number of proteins for a given GO annotation.

### Systemic Effects of Tobacco Smoking in Human Embryogenesis: Redox and Prostaglandin Metabolic Processes

The modularity data gathered from the major PPI-CPI network (**Fig. 1C**) were subjected to GO analysis. The GO analysis of clusters 1, 4, 11, 16, and 20 (**Fig. 3A–E**) revealed five main process annotations: (i) oxidation-reduction (redox), (ii) prostaglandin metabolism, (iii) steroid biosynthesis, (iv) lipid modification, and (v) unsaturated fatty acid metabolism (Tables S4, S7, S14, S19 and S23 in Supporting Information S1). Given the overlap among the different processes, these subnetworks were merged into a single network (**Fig. 3F**). It was observed that lipophilic molecules (e.g., chrysene, toluene, benz[a]anthracene, benzo[b]fluoranthene, 7H-dibenzo(c,g)carbazole, 2-naphthylamine, 4-aminobiphenyl and 5-methylchrysene; Table S2 in Supporting Information S1) were observed to be most connected to the proteins annotated as being involved in redox processes (**Fig. 3A**). Tobacco consumption has been associated with altered redox mechanisms and the generation of oxidative stress, leading to an inflammatory response [31], [32], [33], [34]. In this sense, within the merged network (**Fig. 3F**), two prostaglandin synthases (PTGS1 and PTGS2), and two 5-lipooxygenases (ALOX5 and ALOX15B), which play a role in the synthesis of leukotrienes [35], were identified. PTGSs are not only related to inflammatory responses when they are present at high levels in tissues but are also associated with normal pregnancies due to promoting adequate circulatory adaptation and regular maternal-fetal blood flow [32], [36]. In addition to the results of our GO analyses, it is known that maternal smoke diminishes prostaglandin levels, which causes low birth weight [36]. In addition, arsenic (**Fig. 3B**), which is present in this module, is related to increased oxidative stress via redox mechanisms [37]. Considering the data amassed in this module, it is possible to speculate that pro-oxidative stimulation by TCs, such as those included in **Fig. 3** **,** can generate a pro-inflammatory cascade, followed by downregulation of PTGSs and increased availability leukotriene, which promotes a continuous pro-inflammatory process. To corroborate this information, we used the transcriptomic data available for placenta and cord blood of passive smoking and non-smoking women [11]. In fact, the transcriptomic data analysis of placenta and cord blood of passive smoking women using landscape evaluation of the clusters 1, 4, and 20 (Fig. S1 in Supporting Information S3) indicated that the PTGS and ALOX genes are underexpressed when compared to non-smoking women. Interestingly, almost all glutathione S-transferase genes (e.g., GSTM1, GSTA1), which catalyze the conjugation of reduced glutathione with toxic xenobiotic substrates and confer antioxidative stress protection [38], are also downregulated in the placenta and cord blood of passive smoking women (Fig. S1 in Supporting Information S3), supporting the idea that TCs induce a pro-oxidative condition in embryo.

### Systemic Effects of Tobacco Smoking on Human Embryogenesis: Regulation of Cell Communication and Cell-cell Signaling

Cellular communication is of great importance for embryonic development, being essential to coordinate the different biochemical signals required to control cellular differentiation and migration. Interestingly, GO analysis of clusters 2 and 18 (**Fig. 4**) revealed two related processes: (i) regulation of cell communication and (ii) cell-cell signaling (Tables S5 and S21 in Supporting Information S1). Considering the different proteins found in cluster 2 (**Fig. 4A**), two nodes appear to be important TC targets: (i) signal transducer and activator of transcription 3 (STAT3), which is related to cell-cell signaling in stem cell cultures [39]; and (ii) colony stimulating factor receptor-β (CSF2RB), a CSF2 receptor molecule that is important for post-blastocyst embryonic development, embryo differentiation, and implantation [40]. Epidermal growth factor (EGF) and its receptor EGFR were also present in this subnetwork (**Fig. 4A**). EGFR is a plasma membrane glycoprotein that is necessary for implantation and epithelial differentiation as well as for cell signal transmission during embryogenesis [41], [42]. It should be noted that both EGF and EGFR were linked to cadmium and methylamine (**Fig. 4A**) in our systems chemo-biology data. Other growth factors, such as nerve growth factor (NGF) and transforming growth factor α (TGFA), are also present in cluster 2. It is possible that the selected constituents, cadmium and methylamine (**Fig. 4**), can play a negative role in cell-cell signaling via inhibition of growth factors and its receptors. Considering the transcriptomic data available for the placenta and cord blood of passive smoking women [11], we observed that EGFR gene and other cell-cell signaling-associated genes are downregulated when compared to non-smoking women (Figs. S2 and S3 in Supporting Information S3).

It should be noted that in the major CPI-PPI network, 1,3-butadiene is linked to HOXD13 (**Fig. 1C**), whose mutations are associated with abnormal limb length [43]. Considering that tobacco abuse can lead to limb aberrations in newborns [3], the HOXD cluster should be an interesting target with respect to understanding the effects of cigarette compounds during development. Moreover, cluster 2 (**Fig. 4A**) contains ERBB2, ERBB3 and ERBB4, which are all members of the tyrosine kinase family and show a similar structure to EGFR, which appears to be crucial for skeletal development [44], and are also downregulated in the placenta and cord blood of passive smoking women (Fig. S2 in Supporting Information S3).

### Systemic Effects of Tobacco Smoking in Human Embryogenesis: Metabolism of DNA, DNA Damage Stimulus, the Cell Cycle and Chromatin Organization

In the GO analysis of clusters 3, 6, 17 and 21 (**Fig. 5**), we identified two related processes: (i) RNA-splicing and (ii) metabolism of nucleotides and DNA (Tables S6, S9, S20 and S24 in Supporting Information S1). TCs were found associated with the metabolism of nucleotides in four different clusters, but each cluster contained different interacting compounds, including both hydrophilic (catechol) (**Fig. 5C** **to** **5E**) and lipophilic (chrysene and 1,3-butadiene, crotonaldehyde) (**Fig. 5B**) as well as organic (chrysene and 1,3-butadiene) (**Fig. 5B**) and inorganic (beryllium, polonium-210 and arsenic) substances (**Fig. 5A, 5C, 5D, and 5E**). Remarkably, in cluster 6, these substances are linked to HPRT1 (**Fig. 5B**), a hypoxanthine phosphoribosyltransferase that is responsible for the metabolism of purines [45].

Moreover, 1,3-butadiene, has been found to be linked to increased genotoxic stress due to DNA damage through the formation of DNA-DNA cross-links at adenine and guanine nucleobases by its metabolites, 1,2,3,4-diepoxybutane and 3,4-epoxy-1,2-butanediol [46], [47]. The compound 1,3-butadiene has also been associated with epigenotoxic effects caused by the loss of global DNA methylation and trimethylation of histone H3 lysines 9 and 27 and H4 lysine 20, all of which are known for their roles in regulating gene expression patterns [47].

Next, in the GO analysis of clusters 5, 8 and 9 (**Fig. 6**), we identified two related processes: (i) DNA damage stimulation and (ii) the cell cycle (Tables S8, S11 and S12 in Supporting Information S1). In this cluster, arsenic binds directly to PLM (**Fig. 6B and 6D**), which is a protein with functions involved in chromatin organization, cell differentiation, DNA repair, protein sequestration and post-translational modifications [http://www.genecards.org]. PML is linked to significant proteins that regulate cell cycle such as p53, p300 and BING2 (**Fig. 6B and 6D**). Another TC, urethane, is directly connected to FOS (**Fig. 6B and 6D**), a central protein involved in proliferation, and TNF, a pro-inflammatory cytokine. Urethane is reported to alter placental morphology and down-regulates cell cycle genes as well as cytokines and other growth factors [48]. Interestingly, TCs downregulate the expression of genes associated with the metabolism of nucleotides and DNA, and cell cycle, as observed by transcriptomic analysis (Figs. S4 and S5 in Supporting Information S3).

In the GO analysis of cluster 7 (**Fig. 6B**), we only identified chromatin organization (Table S10 in Supporting Information S1) as a major biological process. Cluster 7 included dimethylamine, which is connected to MBD2 (**Fig. 6B**), a protein associated with regions of methylated DNA in CpG islands that can recruit histone deacetylases (HDACs) and DNA methyltransferases [http://www.genecards.org]. DNA methylation is also correlated with gene silencing through polycomb repression complexes (PRC) [49]. PRC is involved in the silencing of many HOX genes [49], which are critical for normal fetus development. Additionally, MBD2 is correlated with the inactivation of sexual chromosomes and is a candidate for recruiting DNA-methyltransferases (DNMTs) to the silenced promoters of long-term repressed genes [50]. Taking into account the effects of TCs in the expression of genes associated to chromatin remodeling, like HDACs, it can be observed that placenta and cord blood of passive smoking women showed a downregulation of those genes (Fig. S6 in Supporting Information S3), supporting the idea that TCs can affect chromatin remodeling during embryogenesis.

### Effect of Nicotine on Retinoic Acid Signaling, Cell Proliferation and Differentiation

A second analysis using systems chemo-biology tools was developed to elucidate the relationships between nicotine, RA signaling and cell differentiation in the fetus during embryonic development in female smokers. The extracted subnetwork was examined separately due the distinct module involving nicotine and its interacting proteins. RA was added to the network because we observed that many proteins connected to nicotine are related to embryonic development and RA signaling.

Thus, the amassed data allowed the design of a major CPI network associated with nicotine and RA signaling (**Fig. 7**), which revealed several proteins that related to embryonic development, stress responses, and cell proliferation. Several of the proteins in the CPI network are directly connected to nicotine, including (i) VEGFA, a factor that induces blood vessel formation (angiogenesis) [51]; (ii) DNMT1, a DNA methyltransferase responsible for the methylation of 5′CpG islands in DNA (**Fig. 7**) [52]; (iii), FOS and JNK1 (MAPK8), which are both inducers of cell proliferation [53], [54]; and (iv) SOD2, which is responsible for mitochondrial superoxide dismutation. In addition, many proteins involved in cellular responses to stress, DNA damage and inflammation are interconnected with nicotine in the CPI network (**Fig. 7**).

We observed a connection between nicotine and JNK1 through their association with RARα in the CPI network (**Fig. 7**). JNK1 is expressed when the cell undergoes cellular stresses, such as inflammation, oxidative stress, and heat [55]. In a murine model, nicotine was found to be related to the expression of JNK1 in respiratory system tissues through nicotinic receptors and receptor kinins B1 and B2, whose stimulation by bradykinin leads to increased levels of intracellular Ca^2+^ [53]. Cellular stress can activate JNK1, which phosphorylates RARα and causes its proteosomal degradation [55].

Supporting the idea that nicotine can induce the activation of pro-inflammatory cascades and different cellular stress pathways, the placenta and cord blood of passive smoking women showed an upregulation of interleukin receptors (e.g., IL2RA; IL2RB), VEGFA, FOS, JAK1, among others (Fig. S7 in Supporting Information S3). Moreover, genes associated with antioxidative stress, like SOD2, are underexpressed when compared to non-smoking women (Fig. S7 in Supporting Information S3).

**Figure 8:**
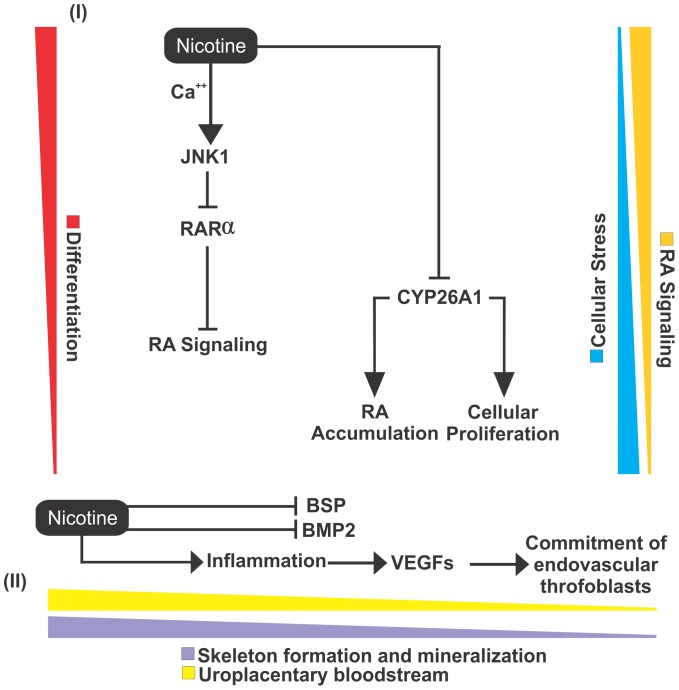
A molecular model illustrating how nicotine could potentially affects differentiation. In the first part of the model (I), it can be observed that by generating cellular stresses, nicotine promotes the recruitment of JNK1 through the influx of intracellular Ca^2+^. JNK1, by itself, promotes the inhibition of RARα. Finally, nicotine promotes the inhibition of CYP26A1, which generates an accumulation of RA in the cell and an increase in cell proliferation. In the second part of the model (II), the inhibition of BMP2 and BSP is promoted by nicotine, which results in the negative regulation of bone mineralization and skeletal development. In addition, nicotine promotes a pro-inflammatory reaction that recruits VEGF and placental growth factors, which leads to an impairment of the endovascular trophoblast, resulting in a fetal growth restriction.

The systems chemo-biology analysis performed in this study also showed that nicotine is directly connected to the protein CYP26A1 (**Fig. 7**), whose coding gene is downregulated in placenta and cord blood of passive smoking women (Fig. S7 in Supporting Information S3). This protein is responsible for regulating RA levels [56] and is expressed in a spatial-temporal manner during the development of mice, mainly in the anterior segment of the embryo and in the neural crest-derived mesenchyme [56]. However, inhibition of this protein generates an accumulation of RA and leads to deformities in the embryo, such as abnormalities in the cerebellum, urogenital tract, and spinal cord [56]. Moreover, nicotine exhibited 22 proteins in common with RA (**Table 2**). These proteins are mostly related to the immune system, stress, and cell proliferation (**Table 2**), indicating that nicotine affects RA signaling through cellular stress caused by constant tobacco use. Interestingly, we observed that nicotine was directly linked with VEGFA in our analysis (**Fig. 7**). Exposure to nicotine could results in an increase in pro-inflammatory signaling, leading to abnormal expression of VEGFA and other placental growth factors, reducing uroplacental blood flow and culminating in fetal growth restriction [57] (**Fig. 8**), an idea that is supported by transcriptomic data (Fig. S7 in Supporting Information S3).

**Table 2:** The relationships between common proteins, nicotine and RA and their specific biochemical functions. These data were obtained from the GeneCards (http://www.genecards.org) and iHop (http://www.ihop-net.org/UniPub/iHOP/) databases.

Protein	Biological function	Role
VEGFA	Growth factor	Crucial role in angiogenesis, vasculogenesis and endothelial growth
TGFB1	Cytokine	Acts in differentiation, proliferation, adhesion and migration; also a potent stimulator of bone growth
ALPP	Alkaline phosphatase	Expressed in the placenta
HSF	Transcription factor	Activated under conditions of heat or other cellular stress
FGF2	Growth factor	Involved in tumor growth, development of the nervous system, cell differentiation and angiogenesis
TH	Hydroxylase	Hydroxylase that functions in the physiology of adrenergic neurons
FOS	Nuclear phosphoprotein	Nuclear phosphoprotein that participates in cell differentiation, proliferation and apoptosis
IL10	Cytokine	Involved in the immune response against pathogens and in the inflammatory response; also related to the intestinal immune system
DNMT1	Methyltransferase	DNA methylation and the establishment of methylation patterns
IL1	Cytokine	Involved in the immune response to pathogens and the inflammatory response
AKT1	Kinase	Involved in tumor formation, angiogenesis and insulin regulation
BAX	Transcription factor	Pro-apoptotic protein
ALPL	Alkaline phosphatase	Mineralization of bone matrix
BCL1 (IL5)	Cytokine	Involved in the immune response against pathogens and the inflammatory response
NOS2A	Nitric oxide synthase	Produces nitric oxide (NO)
PPARG	Proliferator peroxisome receptor	Regulator of adipocyte differentiation and glucose homeostasis
K60 (IL8)	Chemokine	Involved in the inflammatory response; angiogenesis inducer
CREB1	Transcription factor	Controls circadian rhythm, tumor suppressors and the expression of various genes involved in cell survival
PLAU	Protease	Involved in extracellular matrix degradation and possibly tumorigenesis
IL2	Cytokine	Essential in the proliferation of T-cells of the immune system. Stimulates the production of B-cells, monocytes and natural killer cells
KDR (VEGFR)	Growth factor	Plays a crucial role in vasculogenesis and angiogenesis
RARB	Retinoic Acid Receptor	Involved in cell differentiation, cell growth arrest, and signaling and transcription of target genes

### Role of Nicotine in the Differentiation of Bone Tissue

An indirect association of nicotine with RA receptors was observed in the network via the influence of nicotine on the transcription factor JUN (**Fig. 7**). The JUN protein can be activated by the action of JNK1 during osteoblast differentiation [58]. In a smoking woman the blood concentration of nicotine are maintained at a stable level depending on the degree of tobacco use [59]. During embryogenesis, constant levels of nicotine can affect bone development, and morphological data have demonstrated a decrease in bone and cartilage growth [60]. An additional impact of nicotine on bone tissue differentiation involves the relationship with the BMP2 and BSP proteins. The BMP protein family includes the most potent osteogenic growth factors described to date [51] and is connected to nicotine (**Fig. 7**). A study in rabbits showed that treatment with nicotine affects BMP2 RNA levels and the activity of osteoblasts [51]. Similarly, the BSP protein is a glycoprotein that acts on bone mineralization, which has also been described as being inhibited by nicotine in rat osteoblast cells [61]. Corroborating these findings, the transcriptomic data of placenta and cord blood of passive smoking women support the fact that nicotine and other TCs inhibit the expression of BMP2 (Fig. S8 in Supporting Information S3).

### Modularity and Centrality Analyses Linking Nicotine with Abnormal Embryonic Development

Once the CPI network was generated (**Fig. 7**), we aimed to understand which major protein clusters might be present. In this sense, the CPI network (**Fig. 7**) showed the presence of six modules with a coefficient of cohesion greater than or equal to 3.00 (Clusters 1–6, Fig. S3 in Supporting Information S2). It was observed that nicotine appeared in clusters 1–4 (Fig. S3A–D in Supporting Information S2), but not associated with RA (only in Fig. S3C in Supporting Information S2), which exhibits many connections other than nicotine in the network. Nicotine is connected to 49 proteins with 281 connections, and RA is connected to 130 proteins with 1,471 connections (**Fig. 7**). From the systems chemo-biology analysis, it was observed that nicotine more readily clustered in a network focused on proteins involved in development and cellular stress **(** **Fig. 7** **)**. We also observed that certain clusters did not contain either nicotine or RA (Figs. S3E and F in Supporting Information S2). In cluster 5 (Fig. S3E in Supporting Information S2) there are a prevalence of proteins linked to (i) chromatin remodeling, such as EZH2, EED, SUZ12, DNMT1, DNMT3A, DNMT3B, HDAC2, HDAC4, HDAC5, and (ii) development and differentiation, including several HOX proteins [A1, 1C (A5), 4B (D4), 2I (B1), B13, B4 and 4F (A11)], PAX1, PAX6, NANOG, RAR, RXRβ, NOTCH1, CYP2B6, and CYP26A1.

To identify the major nodes within the CPI network (**Fig. 8**), we calculated betweenness, closeness and node degree centralities. From these analyses, two graphs were generated containing the proteins that showed the highest centrality values (Figs. S4 and S5 in Supporting Information S2). Interestingly, these nodes present a similar relevance order in both graphs. Thus, RA, nicotine, JNK1, p300, AKT1, p53 and ERK showed the highest betweenness, closeness and node degree values (Figs. S4 and S5 in Supporting Information S2). As these proteins play major roles in cellular physiology, it was expected that they would exhibit higher values for the three variables. The proteins with the highest values were taken into consideration in the design of a molecular model of the effect of nicotine on embryonic development (**Fig. 8**). In the centrality analysis, it was observed that p300 appeared as an important node, showing the highest values of betweenness, closeness, and the node degree (Figs. S4 and S5 in Supporting Information S2). This scenario demonstrates that there is a major influence of p300 on the network regarding the number of connections with other proteins (92 proteins), the implications of its importance for neighboring proteins (closeness) and its relationships to clusters and bioprocesses (betweenness). Therefore, the negative regulation of this protein induced by nicotine can also lead to fetal malformations and could be a potential study target for understanding the influence of nicotine in development. Noteworthy, the transcriptomic analysis of extraembryonic tissues extracted from pregnant passive smoking women showed a downregulation of p300-coding gene (Fig. S8 in Supporting Information S3).

An important issue that should be addressed in the future is the influence of the major nicotine metabolites on the activity of the enzymes and proteins observed in this work. It has been reported that 70–80% of nicotine is metabolized to cotinine by CYP2A6 to produce nicotine and a cytoplasmic aldehyde oxidase [62]. However, nicotine can generate an elevated number of different metabolites, whose mechanism of action is not clear [62]. Additionally, the mechanism of detoxification of nicotine and cotinine is based on the glucuronidation of both molecules, accounting for 40–60% of the nicotine found in urine [62]. Unfortunately, for the majority of compounds present in tobacco smoke observed in this work, the data about its metabolization or detoxification are virtually unknown. The use of metabolomic techniques associated with systems chemo-biology tools should improve our understanding of how nicotine and other TCs physiologically affect development.

## Conclusions

**Figure 9:**
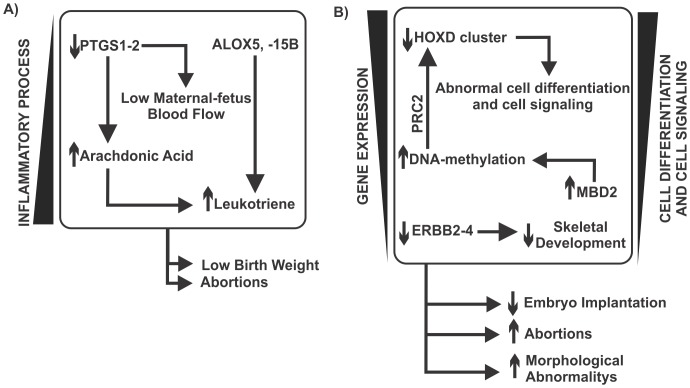
A model of the interactions from a systemic view showing how TCs affect development. In (A), we show that increasing TC levels generate a pro-inflammatory cascade by increasing the levels of PTGS1 and PTGS2. PTGSs are associated with inflammatory responses and are essential for normal pregnancy. Disturbances in PTGS expression could cause impairments in fetal development. TCs are connected to ALOX5 and ALOX15B, which are proteins involved in the synthesis of leukotriene, a molecule that plays pivotal roles in pro-inflammatory responses. The consequence of (A) is low birth weight in newborn infants, abortions and increased proliferation. Moreover, in (B), TCs are linked to BING2 and USP2, which are proteins related to increased activity of MDM2. This MDM2 mediated up-regulation can rapidly down-regulate p53 protein, leaving the cell more susceptible to DNA damage. TCs also down-regulate HPRT1, diminishing purine metabolism. This system exhibits a relationship with increased proliferation. The systems in (C) shows that TCs are associated with the generation of superoxides due to up-regulating NADH oxidase, which increases ROS levels and, consequently, oxidative stress. Increased oxidative stress is known to be related to birth defects. In addition, system (C) is associated with low birth weight and low neutrophil activity. Moreover, (D) shows the relationship between TCs and low hormone synthesis and signaling. Exposure to TCs could have a negative effect on androgen and estrogen solubility due to acting on the UGT cluster. TCs could also be associated with low levels of cholesterol synthesis due to increasing the levels of CYPs and diminishing the levels of FDFT1 and FDPS, which are two enzymes related to cholesterol synthesis. Low cholesterol availability would decrease general hormone synthesis. In addition, TCs affect the transport of cholesterol to the mitochondria by acting on the membrane protein StAR. Finally, in (E), the system shows the relationships between TCs and decreased global gene expression and cellular differentiation and signaling. The activities of the TCs would increase the levels of MBD2, a methylation enzyme. DNA methylation is related to gene silencing. We postulate that TCs could affect the PRC2 complex via its methylation and disturb gene expression, including that of HOX genes. TCs could also have a negative effect on gene expression by increasing YWHAH levels, which would decrease the levels of the master kinase PDPK1 and is linked to AKT activation and SMAD nuclear translocation. In addition, NOTCH signaling could be affected through the action of TCs on APP activation.

In the present study, we showed, using systems chemo-biology tools, how the primary harmful constituents of tobacco interact with specific biological processes and affect them. Our cluster analysis results show that TCs act in many bioprocesses, including cell communication and signaling, hormone synthesis and signaling, DNA metabolism, DNA repair, and inflammation, whose results were supported by landscape network analysis of transcriptomic data of extraembryonic tissues gathered from passive smoking women and non-smoking women. Although these processes have wide effects on cellular and embryonic physiology, they can be disturbed by the levels of the constituents of tobacco smoke. Because these effects are complex, we developed an interaction which comprises two main mechanisms associated with TCs: increased inflammatory processes **(** **Fig. 9A** **)**, and negative regulation of gene expression, cell differentiation and cell signaling (**Fig. 9B**). The systems model is related to low birth weight, an increased probability of abortion, morphological abnormalities (mainly in the skeletal system), low neutrophil activity and increased proliferation rates. Furthermore, our model can help improve knowledge and provide new insights regarding how the chemicals in tobacco cause the many morphological abnormalities observed in the newborn offspring of smoking pregnant women. The role of nicotine in embryonic development has also not been well studied. The analysis performed in this study demonstrates that nicotine has an aggressive effect on cell differentiation, affecting RA signaling in the embryo, inhibiting RA receptors due to intracellular calcium influx and stimulating cell proliferation proteins that antagonize RA activity. Osteoblast differentiation is also affected by nicotine via inhibiting proteins that stimulate bone tissue formation, which complements the TC model. Together, these data show that the birth defects observed in morphological studies could be caused by the negative action of nicotine on RA signaling. The networks also show that the pro-inflammatory pathway triggered by nicotine could be a factor leading to decreased body weight in the fetuses of smoking women. Finally, cluster analysis shows a systemic effect of nicotine, which could affect the network in a more aggressive and short-term way via cellular stress cascades.

## Supporting Information

Supporting Information 1 **Table S1** Transcriptomic data of the proteins directly linked to the selected tobacco constituents (TCs). **Table S2** List of tobacco constituents (TCs) found in the major CPI-PPI network (Fig. 1). The solubility of each compound was accessed using the program ALOGPS 2.1. Those compounds with solubility less than 20 g/l were considered lipophilic. **Table S3** GO processes present in the main tobacco constituents (TCs)-associated CPI-PPI network (Fig. 1). **Table S4** GO processes present in the cluster 1 (Fig. 3A). **Table S5** GO processes present in the cluster 2 (Fig. 4A). **Table S6** GO processes present in the cluster 3 (Fig. 5A). **Table S7** GO processes present in the cluster 4 (Fig. 3B). **Table S8** GO processes present in the cluster 5 (Fig. 6A). **Table S9** GO processes present in the cluster 6 (Fig. 5B). **Table S10** GO processes present in the cluster 7 (Fig. 6B). **Table S11** GO processes present in the cluster 8 (Fig. 6C). **Table S12** GO processes present in the cluster 9 (Fig. 6D). **Table S13** GO processes present in the cluster 10 (S-Fig. 2A). **Table S14** GO processes present in the cluster 11 (Fig. 3C). **Table S15** GO processes present in the cluster 12 (S-Fig. 2B). **Table S16** GO processes present in the cluster 13 (S-Fig. 2C). **Table S17** GO processes present in the cluster 14 (S-Fig. 2D). **Table S18** GO processes present in the cluster 15 (S-Fig. 2E). **Table S19** GO processes present in the cluster 16 (Fig. 3D). **Table S20** GO processes present in the cluster 17 (Fig. 5C). **Table S21** GO processes present in the cluster 18 (Fig. 4B). **Table S22** GO processes present in the cluster 19 (S-Fig. 2F). **Table S23** GO processes present in the cluster 20 (Fig. 3E). **Table S24** GO processes present in the cluster 21 (Fig. 5D). **Table S25** GO processes present in the cluster 22 (S-Fig. 2G).
(XLSX)

Supporting Information 2 **Figure S1** Graph showing the relationship of closeness and betweenness of the TCs in the major CPI-PPI network. All nodes in the graph present a mean above average in both closeness and betweenness. The color represents the soluble property of the TCs (Light blue = hydrophilic and Yellow = lipophilic). Three nodes have distinct color/shape, since they shared a color with the adjacent node [Chromium = Large width node (black); Cadmium = Diamond shape/blue colored; and 7H-dibenzo[cg]carbazole = Orange node]. **Figure S2** Clusters excluded from the analysis due lack of literature data associated with TCs and their given GO, therefore, being highly speculative. In (A), Cluster 10 is composed by 12 nodes and 39 edges, with C*i* = 3,250. The associated hydrophilic component is furfural. Related GO: Glucose Catabolic Process and Pentose-Phosphate Shunt. Cluster 12 (B) is composed by 16 nodes and 43 edges, with C*i* = 2,750. The associated hydrophilic components are cadmium and acrynolitryle. Related GO: Antigen Processing and Presentation. Cluster 13 (C) is composed by 18 nodes and 48 edges, with C*i* = 2,667. The associated hydrophilic component is urethane and the lipophilic is xylene. Related GO: G-Protein Coupled Receptor Protein Signaling Pathway. Cluster 14 (D) is composed by 42 nodes and 109 edges, with C*i* = 2,595. The associated hydrophilic components are hydrazine, resorcinol, nickel and chromium. Related GO: Regulation of Insulin Signaling Pathway. Cluster 15 (E) is composed by 22 nodes and 55 edges, with C*i* = 2,250. The associated hydrophilic components are chromium and acrynolitryle. Whereas the lipophic are xylene, chrysene, 5-methylcrysene, benz[a]anthracene and benzo[b]fluoracene. Related GO: Response to Chemical Stimuli. Cluster 19 (F) is composed by 12 nodes and 27 edges, with C*i* = 2,250. The associated hydrophilic component is lead. Related GO: I-KappaB Kinase/NF-KappaB Cascade. Cluster 22 (G) is composed by 20 nodes and 43 edges, with C*i* = 2,150. The associated hydrophilic components are cadmium, lead, pyrrole and arsenic. Related GO: Heme Biosynthetic Process. **Figure S3** Clusters 1 to 6, extracted from the nicotine CPI-PPI network by MCODE. The blue node is RA and the green node is nicotine. Cluster 1 (A) is composed by 159 nodes and 2373 edges, with C*i* = 14, 925; Cluster 2 (B) is composed by 227 nodes and 2649 edges, with C*i* = 11,670; Cluster 3 (C) is composed by 207 nodes and 1793 edges, with C*i* = 8,662; Cluster 4 (D) is composed by 174 nodes and 1002 edges, with C*i* = 5,759; Cluster 5 (E) is composed by 89 nodes and 300 edges, with C*i* = 3,371; Cluster 6 (F) is composed by 12 nodes and 36 edges, with C*i* = 3,000. Nicotine appears in four clusters (A to D), whereas RA only in C, showing that nicotine is more easily clustered. **Figure S4** Graph showing the relationship of node degree (ND) and betweenness (BT) using all proteins in the nicotine CPI-PPI network. The seven most significant proteins were selected (which are present near the value of 5.0×10^3^). The dotted line shows the threshold of significance, and the values above the line are considered more relevant. **Figure S5** Graph showing the relationship of closeness (CL) and betweenness (BT) from all proteins in the nicotine CPPI-PPI network. The seven most significant proteins were selected (which are present near the value of 5.0×10^3^). The dotted line shows the threshold of significance, and the values above the line are considered more relevant.
(DOCX)

Supporting Information 3 **Figure S1** Network representation of cluster 1,4, and 20 obtained from STRING metasearch engine (A). This network was used for two-state landscape analysis of gene expression (B). Coordinates (X- and Y-axis) represent normalized values of the input network topology. Color gradient (Z-axis) represents the relative gene functional state mapped onto network according to the transcriptomic data input of GSE30032 series file [placenta plus cord blood transcriptomic data from passive smoking women (a) versus placenta plus cord blood from non-smoking women (b)]. In this sense, the mathematical equation z = a/(a+b) was used to calculated the relative gene functional state of condition (a) and condition (b). Thus, the gene expression in condition (a) is greater than condition (b) when z >0.55 (yellow to red colors), lower than (b) when z <0.45 (cyan to blue colors) and equivalent to (b) when 0.45< z <0.55 (green color). The landscape was generated by ViaComplex 1.0 software with the following options: plot as “3D-Graph”, build on “node”, resolution “level-50”, contrast “level-50”, smoothness “level-50” and zoom “level-50”. **Figure S2** Network representation of cluster 2 obtained from STRING metasearch engine (A). This network was used for two-state landscape analysis of gene expression (B). Coordinates (X- and Y-axis) represent normalized values of the input network topology. Color gradient (Z-axis) represents the relative gene functional state mapped onto network according to the transcriptomic data input of GSE30032 series file [placenta plus cord blood transcriptomic data from passive smoking women (a) versus placenta plus cord blood from non-smoking women (b)]. In this sense, the mathematical equation z = a/(a+b) was used to calculated the relative gene functional state of condition (a) and condition (b). Thus, the gene expression in condition (a) is greater than condition (b) when z >0.55 (yellow to red colors), lower than (b) when z <0.45 (cyan to blue colors) and equivalent to (b) when 0.45< z <0.55 (green color). The landscape was generated by ViaComplex 1.0 software with the following options: plot as “3D-Graph”, build on “node”, resolution “level-50”, contrast “level-50”, smoothness “level-50” and zoom “level-50”. **Figure S3** Network representation of cluster 18 obtained from STRING metasearch engine (A). This network was used for two-state landscape analysis of gene expression (B). Coordinates (X- and Y-axis) represent normalized values of the input network topology. Color gradient (Z-axis) represents the relative gene functional state mapped onto network according to the transcriptomic data input of GSE30032 series file [placenta plus cord blood transcriptomic data from passive smoking women (a) versus placenta plus cord blood from non-smoking women (b)]. In this sense, the mathematical equation z = a/(a+b) was used to calculated the relative gene functional state of condition (a) and condition (b). Thus, the gene expression in condition (a) is greater than condition (b) when z >0.55 (yellow to red colors), lower than (b) when z <0.45 (cyan to blue colors) and equivalent to (b) when 0.45< z <0.55 (green color). The landscape was generated by ViaComplex 1.0 software with the following options: plot as “3D-Graph”, build on “node”, resolution “level-50”, contrast “level-50”, smoothness “level-50” and zoom “level-50”. **Figure S4** Network representation of cluster 3, 11 and 21 obtained from STRING metasearch engine (A). This network was used for two-state landscape analysis of gene expression (B). Coordinates (X- and Y-axis) represent normalized values of the input network topology. Color gradient (Z-axis) represents the relative gene functional state mapped onto network according to the transcriptomic data input of GSE30032 series file [placenta plus cord blood transcriptomic data from passive smoker women (a) versus placenta plus cord blood from non-smoker women (b)]. In this sense, the mathematical equation z = a/(a+b) was used to calculated the relative gene functional state of condition (a) and condition (b). Thus, the gene expression in condition (a) is greater than condition (b) when z >0.55 (yellow to red colors), lower than (b) when z <0.45 (cyan to blue colors) and equivalent to (b) when 0.45< z <0.55 (green color). The landscape was generated by ViaComplex 1.0 software with the following options: plot as “3D-Graph”, build on “node”, resolution “level-50”, contrast “level-50”, smoothness “level-50” and zoom “level-50”. **Figure S5** Network representation of cluster 5, 8 and 9 obtained from STRING metasearch engine (A). This network was used for two-state landscape analysis of gene expression (B). Coordinates (X- and Y-axis) represent normalized values of the input network topology. Color gradient (Z-axis) represents the relative gene functional state mapped onto network according to the transcriptomic data input of GSE30032 series file [placenta plus cord blood transcriptomic data from passive smoker women (a) versus placenta plus cord blood from non-smoker women (b)]. In this sense, the mathematical equation z = a/(a+b) was used to calculated the relative gene functional state of condition (a) and condition (b). Thus, the gene expression in condition (a) is greater than condition (b) when z >0.55 (yellow to red colors), lower than (b) when z <0.45 (cyan to blue colors) and equivalent to (b) when 0.45< z <0.55 (green color). The landscape was generated by ViaComplex 1.0 software with the following options: plot as “3D-Graph”, build on “node”, resolution “level-50”, contrast “level-50”, smoothness “level-50” and zoom “level-50”. **Figure S6** Network representation of cluster 7 obtained from STRING metasearch engine (A). This network was used for two-state landscape analysis of gene expression (B). Coordinates (X- and Y-axis) represent normalized values of the input network topology. Color gradient (Z-axis) represents the relative gene functional state mapped onto network according to the transcriptomic data input of GSE30032 series file [placenta plus cord blood transcriptomic data from passive smoker women (a) versus placenta plus cord blood from non-smoker women (b)]. In this sense, the mathematical equation z = a/(a+b) was used to calculated the relative gene functional state of condition (a) and condition (b). Thus, the gene expression in condition (a) is greater than condition (b) when z >0.55 (yellow to red colors), lower than (b) when z <0.45 (cyan to blue colors) and equivalent to (b) when 0.45< z <0.55 (green color). The landscape was generated by ViaComplex 1.0 software with the following options: plot as “3D-Graph”, build on “node”, resolution “level-50”, contrast “level-50”, smoothness “level-50” and zoom “level-50”. **Figure S7** Nicotine-associated network obtained from STRING metasearch engine (A). This network was used for two-state landscape analysis of gene expression (B). Coordinates (X- and Y-axis) represent normalized values of the input network topology. Color gradient (Z-axis) represents the relative gene functional state mapped onto network according to the transcriptomic data input of GSE30032 series file [placenta plus cord blood transcriptomic data from passive smoker women (a) versus placenta plus cord blood from non-smoker women (b)]. In this sense, the mathematical equation z = a/(a+b) was used to calculated the relative gene functional state of condition (a) and condition (b). Thus, the gene expression in condition (a) is greater than condition (b) when z >0.55 (yellow to red colors), lower than (b) when z <0.45 (cyan to blue colors) and equivalent to (b) when 0.45< z <0.55 (green color). The landscape was generated by ViaComplex 1.0 software with the following options: plot as “3D-Graph”, build on “node”, resolution “level-50”, contrast “level-50”, smoothness “level-50” and zoom “level-50”. **Figure S8** Retinoic acid-associated network obtained from STRING metasearch engine (A). This network was used for two-state landscape analysis of gene expression (B). Coordinates (X- and Y-axis) represent normalized values of the input network topology. Color gradient (Z-axis) represents the relative gene functional state mapped onto network according to the transcriptomic data input of GSE30032 series file [placenta plus cord blood transcriptomic data from passive smoker women (a) versus placenta plus cord blood from non-smoker women (b)]. In this sense, the mathematical equation z = a/(a+b) was used to calculated the relative gene functional state of condition (a) and condition (b). Thus, the gene expression in condition (a) is greater than condition (b) when z >0.55 (yellow to red colors), lower than (b) when z <0.45 (cyan to blue colors) and equivalent to (b) when 0.45< z <0.55 (green color). The landscape was generated by ViaComplex 1.0 software with the following options: plot as “3D-Graph”, build on “node”, resolution “level-50”, contrast “level-50”, smoothness “level-50” and zoom “level-50”.
(DOCX)
