# Supplementary material for: Toxicological Effects of the Different Substances in Tobacco Smoke on Human Embryonic Development by a Systems Chemo-Biology Approach
Source: PLoS One. 2013 Apr 29;8(4):e61743. doi: 10.1371/journal.pone.0061743 (PMC3639264; doi:10.1371/journal.pone.0061743)
Supplement: Supporting Information 2 — Figure S1 Graph showing the relationship of closeness and betweenness of the TCs in the major CPI-PPI network. All nodes in the graph present a mean above average in both closeness and betweenness. The color represents the soluble property of the TCs (Light blue = hydrophilic and Yellow = lipophilic). Three nodes have distinct color/shape, since they shared a color with the adjacent node [Chromium = Large width node (black); Cadmium = Diamond shape/blue colored; and 7H-dibenzo[cg]carbazole = Orange node]. Figure S2 Clusters excluded from the analysis due lack of literature data associated with TCs and their given GO, therefore, being highly speculative. In (A), Cluster 10 is composed by 12 nodes and 39 edges, with Ci = 3,250. The associated hydrophilic component is furfural. Related GO: Glucose Catabolic Process and Pentose-Phosphate Shunt. Cluster 12 (B) is composed by 16 nodes and 43 edges, with Ci = 2,750. The associated hydrophilic components are cadmium and acrynolitryle. Related GO: Antigen Processing and Presentation. Cluster 13 (C) is composed by 18 nodes and 48 edges, with Ci = 2,667. The associated hydrophilic component is urethane and the lipophilic is xylene. Related GO: G-Protein Coupled Receptor Protein Signaling Pathway. Cluster 14 (D) is composed by 42 nodes and 109 edges, with Ci = 2,595. The associated hydrophilic components are hydrazine, resorcinol, nickel and chromium. Related GO: Regulation of Insulin Signaling Pathway. Cluster 15 (E) is composed by 22 nodes and 55 edges, with Ci = 2,250. The associated hydrophilic components are chromium and acrynolitryle. Whereas the lipophic are xylene, chrysene, 5-methylcrysene, benz[a]anthracene and benzo[b]fluoracene. Related GO: Response to Chemical Stimuli. Cluster 19 (F) is composed by 12 nodes and 27 edges, with Ci = 2,250. The associated hydrophilic component is lead. Related GO: I-KappaB Kinase/NF-KappaB Cascade. Cluster 22 (G) is composed by 20 nodes and 43 edges, with Ci = 2,150. The associated hy [file pone.0061743.s002.docx]

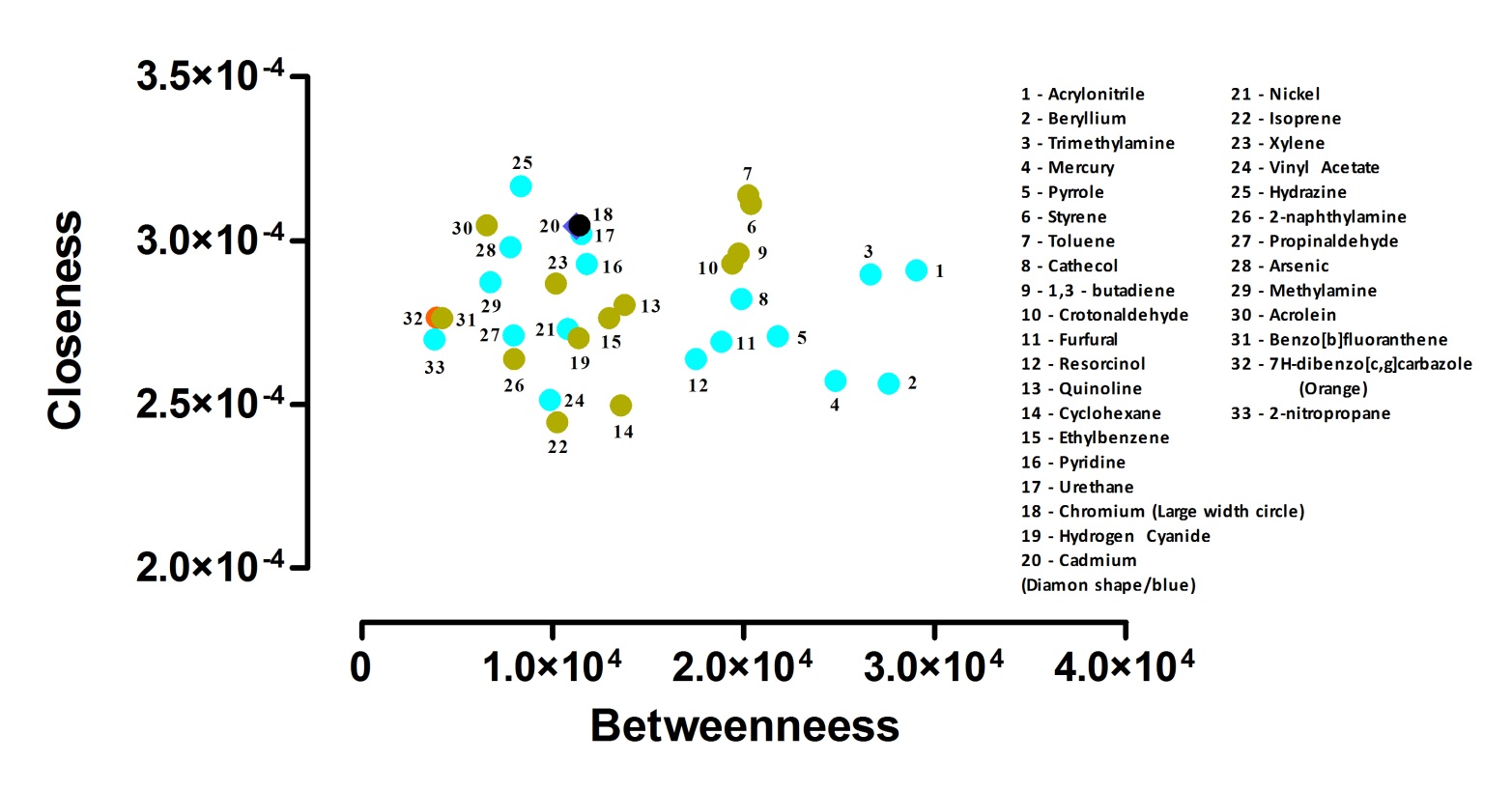


**Figure S1:** Graph showing the relationship of closeness and betweenness of the TCs in the major CPI-PPI network. All nodes in the graph present a mean above average in both closeness and betweenness. The color represents the soluble property of the TCs (Light blue = hydrophilic and Yellow = lipophilic). Three nodes have distinct color/shape, since they shared a color with the adjacent node [Chromium = Large width node (black); Cadmium = Diamond shape/blue colored; and 7H-dibenzo[cg]carbazole = Orange node].


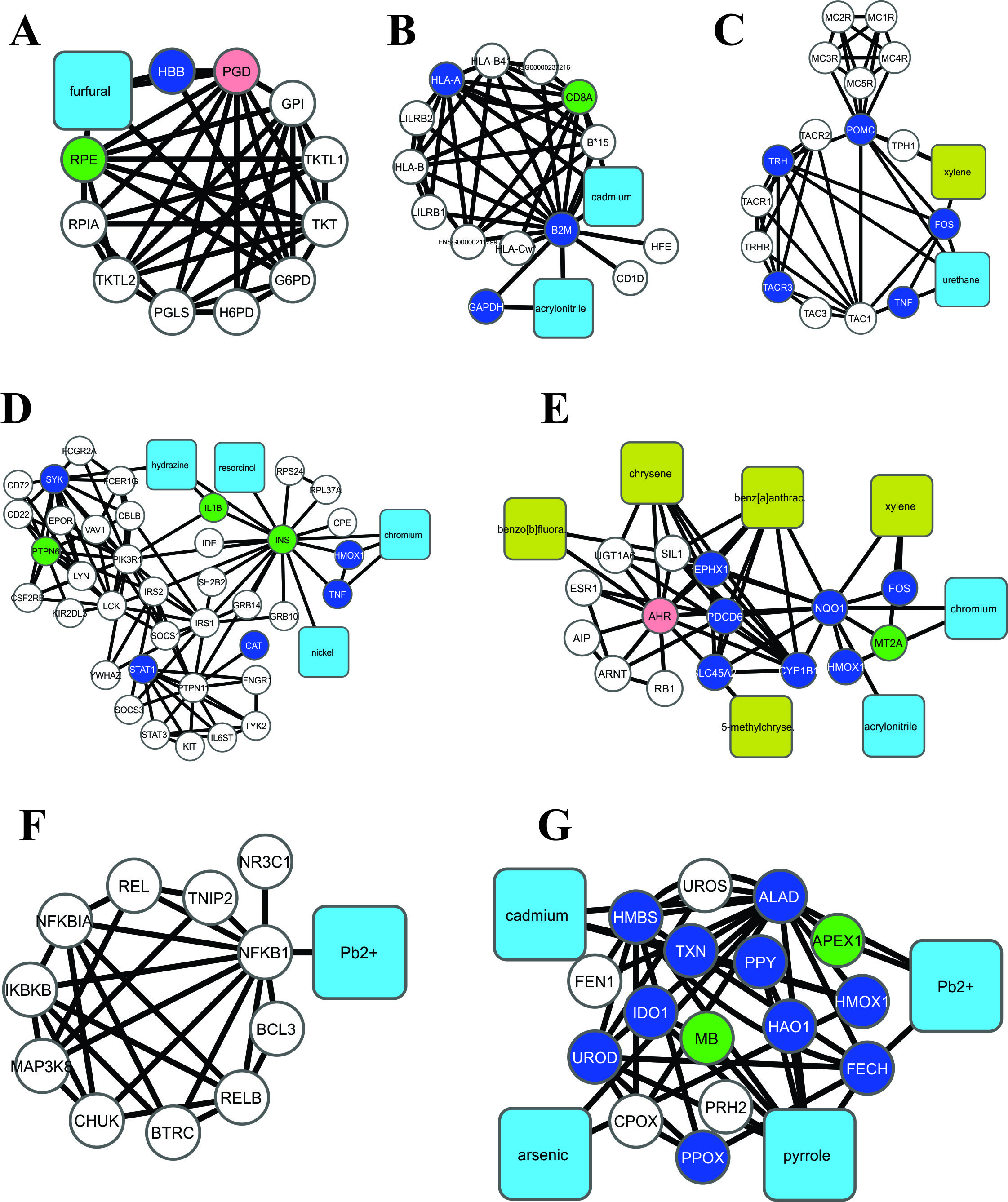


**Figure S2:** Clusters excluded from the analysis due lack of literature data associated with TCs and their given GO, therefore, being highly speculative. In (A), Cluster 10 is composed by 12 nodes and 39 edges, with C*i* = 3,250. The associated hydrophilic component is furfural. Related GO: Glucose Catabolic Process and Pentose-Phosphate Shunt. Cluster 12 (B) is composed by 16 nodes and 43 edges, with C*i* = 2,750. The associated hydrophilic components are cadmium and acrynolitryle. Related GO: Antigen Processing and Presentation. Cluster 13 (C) is composed by 18 nodes and 48 edges, with C*i* = 2,667. The associated hydrophilic component is urethane and the lipophilic is xylene. Related GO: G-Protein Coupled Receptor Protein Signaling Pathway. Cluster 14 (D) is composed by 42 nodes and 109 edges, with C*i* = 2,595. The associated hydrophilic components are hydrazine, resorcinol, nickel and chromium. Related GO: Regulation of Insulin Signaling Pathway. Cluster 15 (E) is composed by 22 nodes and 55 edges, with C*i* = 2,250. The associated hydrophilic components are chromium and acrynolitryle. Whereas the lipophic are xylene, chrysene, 5-methylcrysene, benz[a]anthracene and benzo[b]fluoracene. Related GO: Response to Chemical Stimuli. Cluster 19 (F) is composed by 12 nodes and 27 edges, with C*i* = 2,250. The associated hydrophilic component is lead. Related GO: I-KappaB Kinase/NF-KappaB Cascade. Cluster 22 (G) is composed by 20 nodes and 43 edges, with C*i* = 2,150. The associated hydrophilic components are cadmium, lead, pyrrole and arsenic. Related GO: Heme Biosynthetic Process.


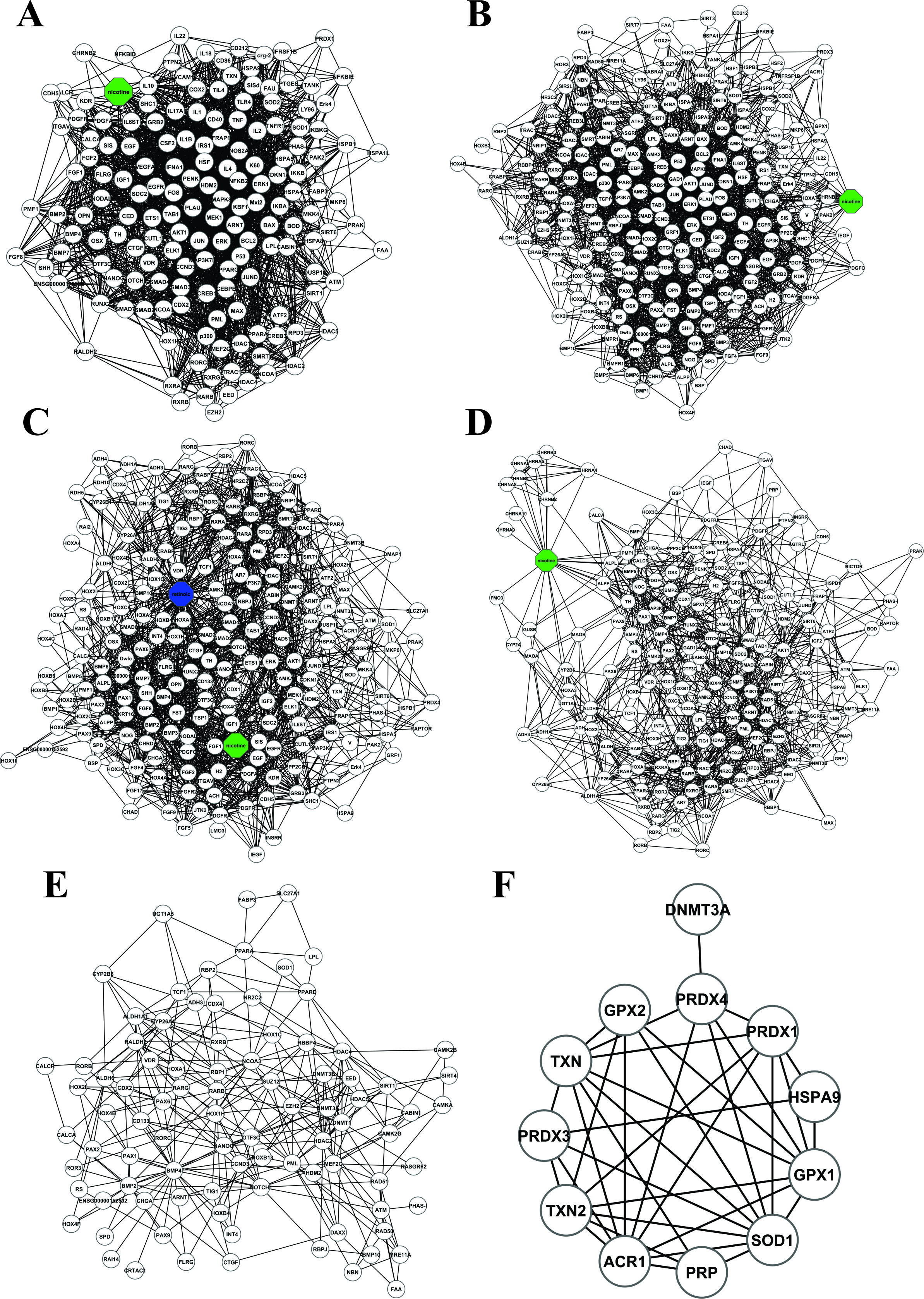


**Figure S3:** Clusters 1 to 6, extracted from the nicotine CPI-PPI network by MCODE. The blue node is RA and the green node is nicotine. Cluster 1 (A) is composed by 159 nodes and 2373 edges, with C*i* = 14, 925; Cluster 2 (B) is composed by 227 nodes and 2649 edges, with C*i* = 11,670; Cluster 3 (C) is composed by 207 nodes and 1793 edges, with C*i* = 8,662; Cluster 4 (D) is composed by 174 nodes and 1002 edges, with C*i* = 5,759; Cluster 5 (E) is composed by 89 nodes and 300 edges, with C*i* = 3,371; Cluster 6 (F) is composed by 12 nodes and 36 edges, with C*i* = 3,000. Nicotine appears in four clusters (A to D), whereas RA only in C, showing that nicotine is more easily clustered.


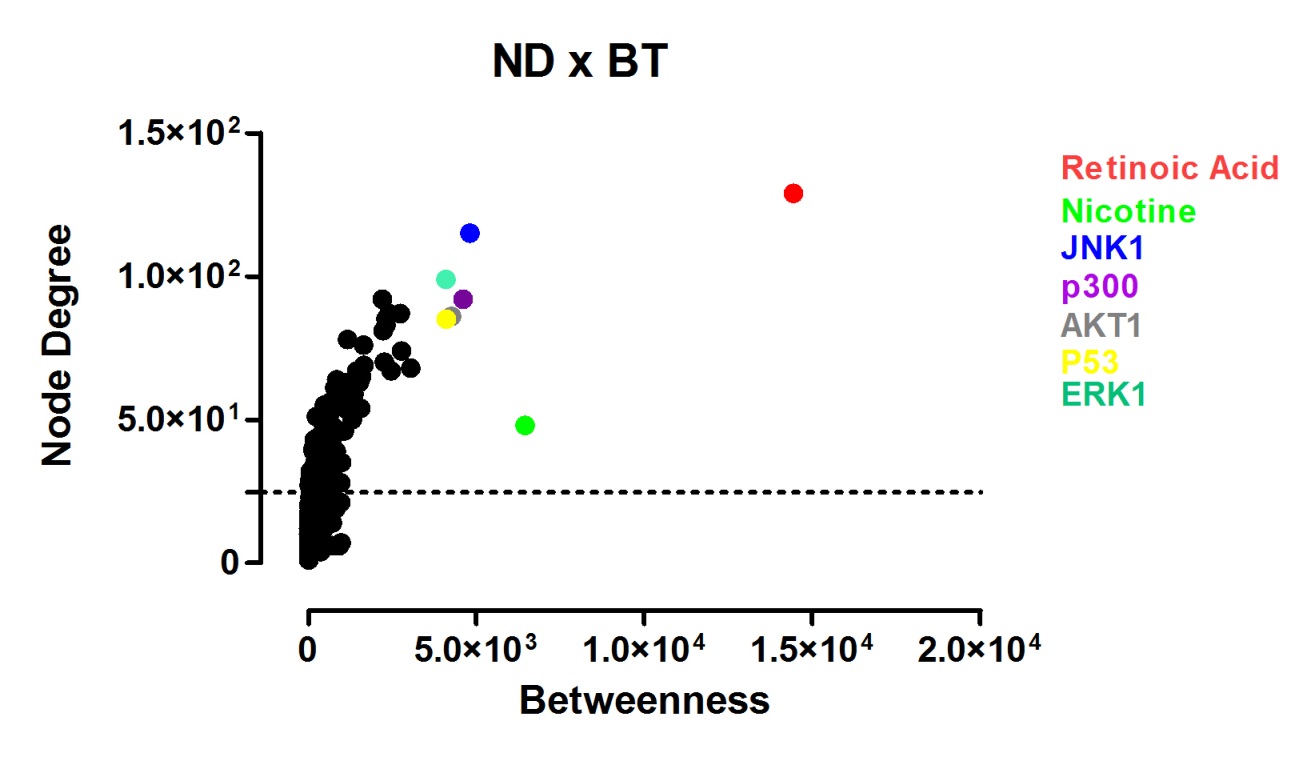


**Figure S4:** Graph showing the relationship of node degree (ND) and betweenness (BT) using all proteins in the nicotine CPI-PPI netowrk. The seven most significant proteins were selected (which are present near the value of 5.0×10^3^). The dotted line shows the threshold of significance, and the values ​​above the line are considered more relevant.


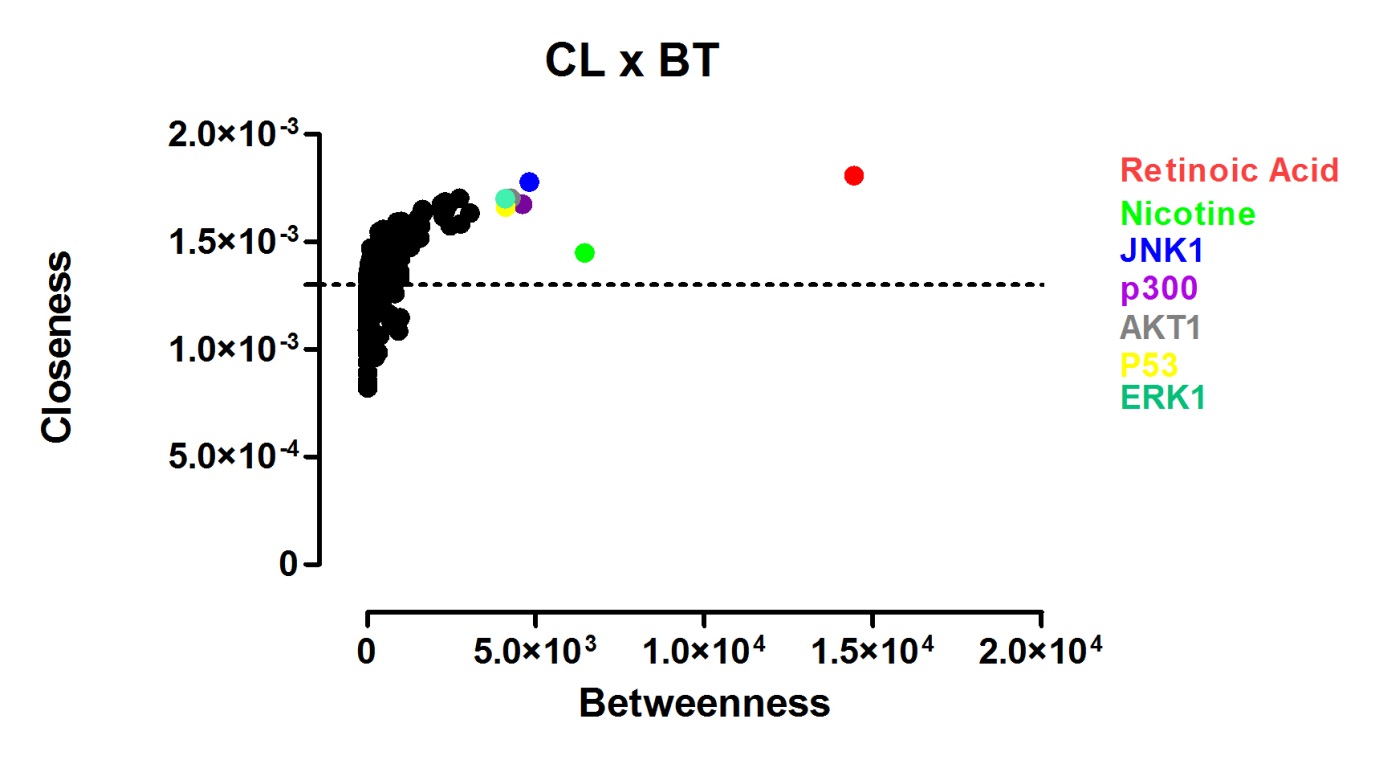


**Figure S5:** Graph showing the relationship of closeness (CL) and betweenness (BT) from all proteins in the nicotine CPPI-PPI network. The seven most significant proteins were selected (which are present near the value of 5.0×10^3^). The dotted line shows the threshold of significance, and the values ​​above the line are considered more relevant.
