# Supplementary material for: Toxicological Effects of the Different Substances in Tobacco Smoke on Human Embryonic Development by a Systems Chemo-Biology Approach
Source: PLoS One. 2013 Apr 29;8(4):e61743. doi: 10.1371/journal.pone.0061743 (PMC3639264; doi:10.1371/journal.pone.0061743)
Supplement: Supporting Information 3 — Figure S1 Network representation of cluster 1,4, and 20 obtained from STRING metasearch engine (A). This network was used for two-state landscape analysis of gene expression (B). Coordinates (X- and Y-axis) represent normalized values of the input network topology. Color gradient (Z-axis) represents the relative gene functional state mapped onto network according to the transcriptomic data input of GSE30032 series file [placenta plus cord blood transcriptomic data from passive smoking women (a) versus placenta plus cord blood from non-smoking women (b)]. In this sense, the mathematical equation z = a/(a+b) was used to calculated the relative gene functional state of condition (a) and condition (b). Thus, the gene expression in condition (a) is greater than condition (b) when z >0.55 (yellow to red colors), lower than (b) when z <0.45 (cyan to blue colors) and equivalent to (b) when 0.45< z <0.55 (green color). The landscape was generated by ViaComplex 1.0 software with the following options: plot as “3D-Graph”, build on “node”, resolution “level-50”, contrast “level-50”, smoothness “level-50” and zoom “level-50”. Figure S2 Network representation of cluster 2 obtained from STRING metasearch engine (A). This network was used for two-state landscape analysis of gene expression (B). Coordinates (X- and Y-axis) represent normalized values of the input network topology. Color gradient (Z-axis) represents the relative gene functional state mapped onto network according to the transcriptomic data input of GSE30032 series file [placenta plus cord blood transcriptomic data from passive smoking women (a) versus placenta plus cord blood from non-smoking women (b)]. In this sense, the mathematical equation z = a/(a+b) was used to calculated the relative gene functional state of condition (a) and condition (b). Thus, the gene expression in condition (a) is greater than condition (b) when z >0.55 (yellow to red colors), lower than (b) when z <0.45 (cyan to blue colors) and equival [file pone.0061743.s003.docx]

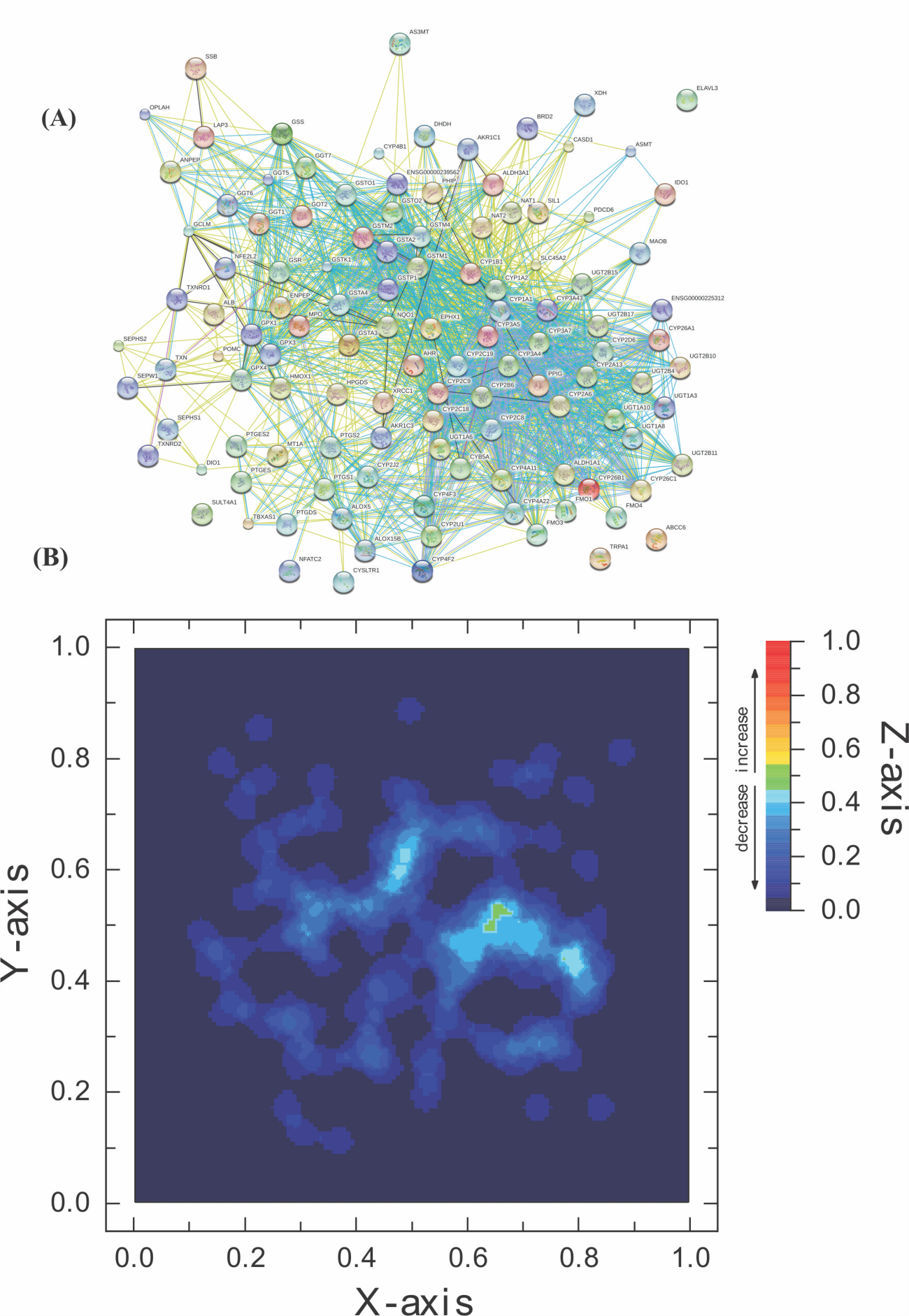


**Figure S1.** Network representation of cluster 1,4, and 20 obtained from STRING metasearch engine (A). This network was used for two-state landscape analysis of gene expression (B). Coordinates (X- and Y-axis) represent normalized values of the input network topology. Color gradient (Z-axis) represents the relative gene functional state mapped onto network according to the transcriptomic data input of GSE30032 series file [placenta plus cord blood transcriptomic data from passive smoking women (a) versus placenta plus cord blood from non-smoking women (b)]. In this sense, the mathematical equation z = a/(a+b) was used to calculated the relative gene functional state of condition (a) and condition (b). Thus, the gene expression in condition (a) is greater than condition (b) when z > 0.55 (yellow to red colors), lower than (b) when z < 0.45 (cyan to blue colors) and equivalent to (b) when 0.45 < z < 0.55 (green color). The landscape was generated by ViaComplex 1.0 software with the following options: plot as "3D-Graph", build on "node", resolution "level-50", contrast "level-50", smoothness "level-50" and zoom "level-50".


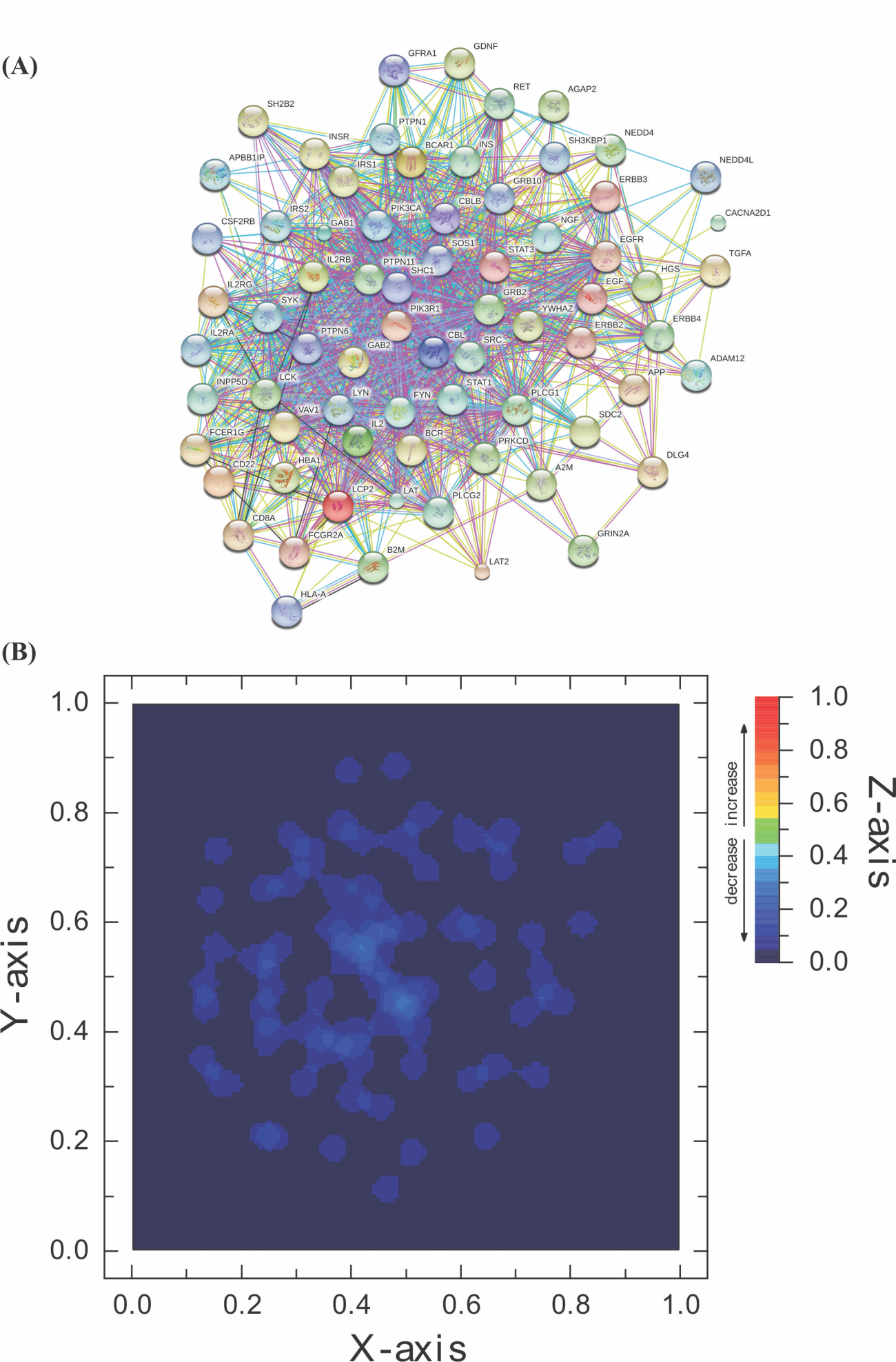


**Figure S2.** Network representation of cluster 2 obtained from STRING metasearch engine (A). This network was used for two-state landscape analysis of gene expression (B). Coordinates (X- and Y-axis) represent normalized values of the input network topology. Color gradient (Z-axis) represents the relative gene functional state mapped onto network according to the transcriptomic data input of GSE30032 series file [placenta plus cord blood transcriptomic data from passive smoking women (a) versus placenta plus cord blood from non-smoking women (b)]. In this sense, the mathematical equation z = a/(a+b) was used to calculated the relative gene functional state of condition (a) and condition (b). Thus, the gene expression in condition (a) is greater than condition (b) when z > 0.55 (yellow to red colors), lower than (b) when z < 0.45 (cyan to blue colors) and equivalent to (b) when 0.45 < z < 0.55 (green color). The landscape was generated by ViaComplex 1.0 software with the following options: plot as "3D-Graph", build on "node", resolution "level-50", contrast "level-50", smoothness "level-50" and zoom "level-50".


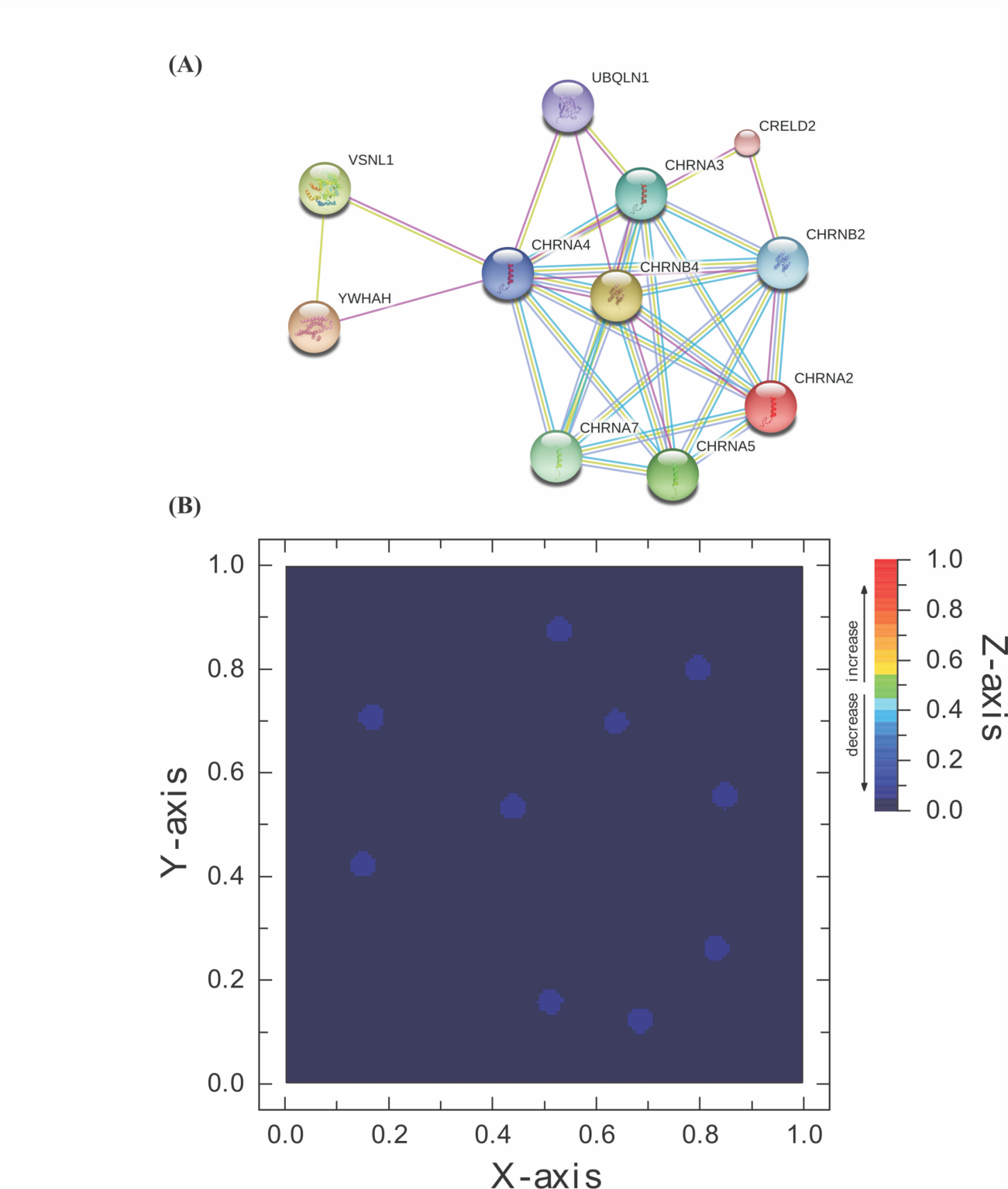


**Figure S3.** Network representation of cluster 18 obtained from STRING metasearch engine (A). This network was used for two-state landscape analysis of gene expression (B). Coordinates (X- and Y-axis) represent normalized values of the input network topology. Color gradient (Z-axis) represents the relative gene functional state mapped onto network according to the transcriptomic data input of GSE30032 series file [placenta plus cord blood transcriptomic data from passive smoking women (a) versus placenta plus cord blood from non-smoking women (b)]. In this sense, the mathematical equation z = a/(a+b) was used to calculated the relative gene functional state of condition (a) and condition (b). Thus, the gene expression in condition (a) is greater than condition (b) when z > 0.55 (yellow to red colors), lower than (b) when z < 0.45 (cyan to blue colors) and equivalent to (b) when 0.45 < z < 0.55 (green color). The landscape was generated by ViaComplex 1.0 software with the following options: plot as "3D-Graph", build on "node", resolution "level-50", contrast "level-50", smoothness "level-50" and zoom "level-50".


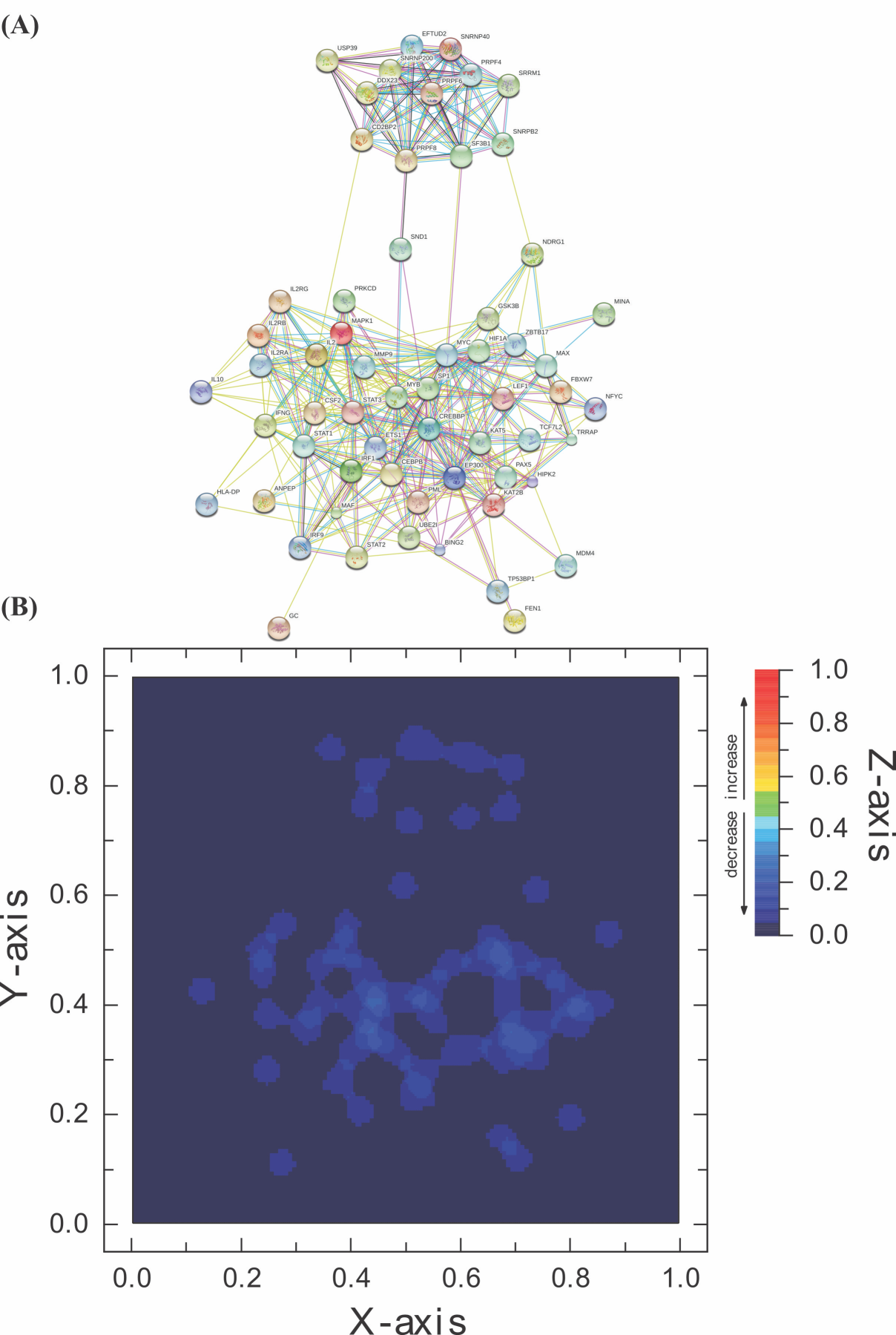


**Figure S4.** Network representation of cluster 3, 11 and 21 obtained from STRING metasearch engine (A). This network was used for two-state landscape analysis of gene expression (B). Coordinates (X- and Y-axis) represent normalized values of the input network topology. Color gradient (Z-axis) represents the relative gene functional state mapped onto network according to the transcriptomic data input of GSE30032 series file [placenta plus cord blood transcriptomic data from passive smoker women (a) versus placenta plus cord blood from non-smoker women (b)]. In this sense, the mathematical equation z = a/(a+b) was used to calculated the relative gene functional state of condition (a) and condition (b). Thus, the gene expression in condition (a) is greater than condition (b) when z > 0.55 (yellow to red colors), lower than (b) when z < 0.45 (cyan to blue colors) and equivalent to (b) when 0.45 < z < 0.55 (green color). The landscape was generated by ViaComplex 1.0 software with the following options: plot as "3D-Graph", build on "node", resolution "level-50", contrast "level-50", smoothness "level-50" and zoom "level-50".


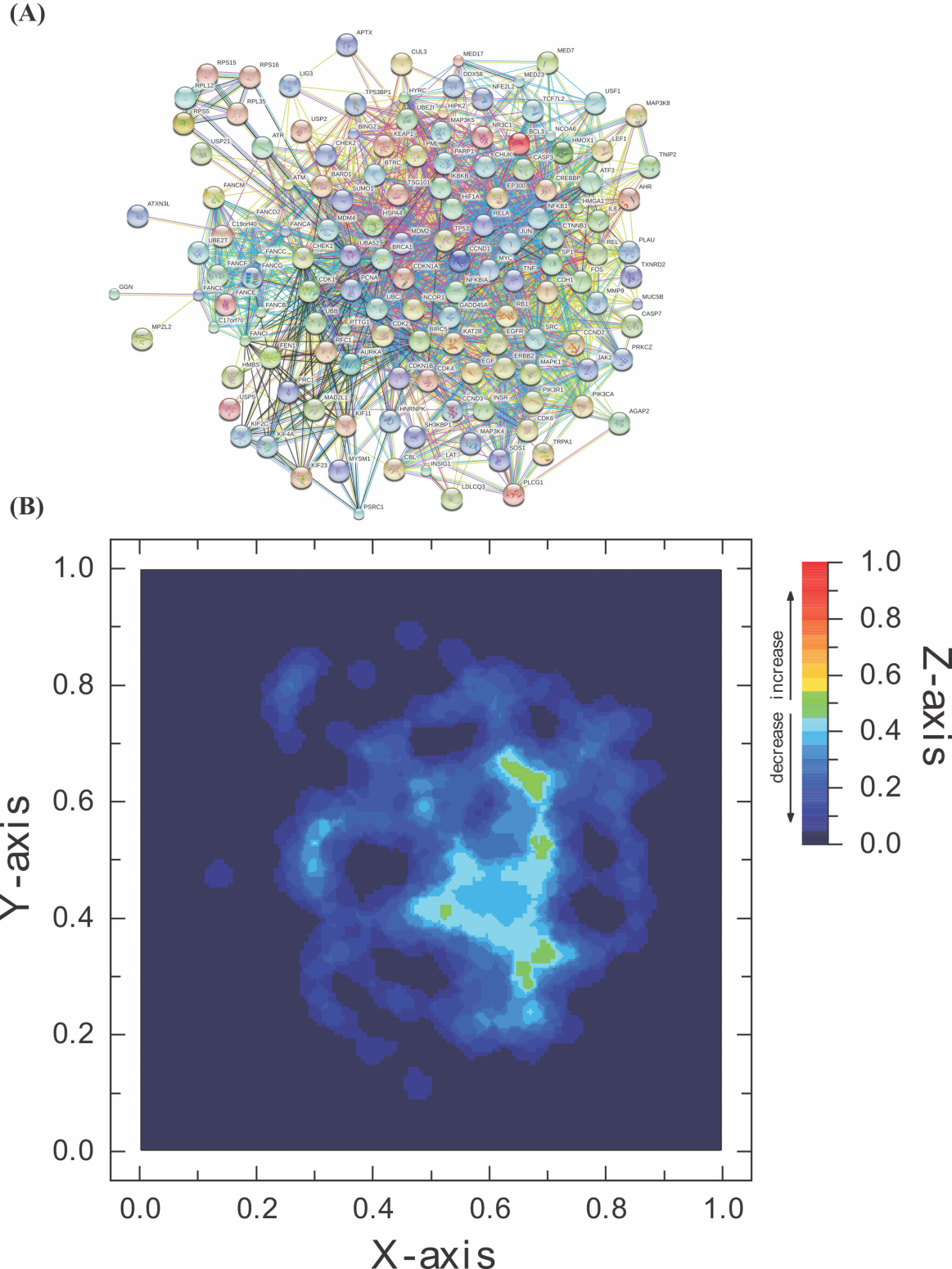


**Figure S5.** Network representation of cluster 5, 8 and 9 obtained from STRING metasearch engine (A). This network was used for two-state landscape analysis of gene expression (B). Coordinates (X- and Y-axis) represent normalized values of the input network topology. Color gradient (Z-axis) represents the relative gene functional state mapped onto network according to the transcriptomic data input of GSE30032 series file [placenta plus cord blood transcriptomic data from passive smoker women (a) versus placenta plus cord blood from non-smoker women (b)]. In this sense, the mathematical equation z = a/(a+b) was used to calculated the relative gene functional state of condition (a) and condition (b). Thus, the gene expression in condition (a) is greater than condition (b) when z > 0.55 (yellow to red colors), lower than (b) when z < 0.45 (cyan to blue colors) and equivalent to (b) when 0.45 < z < 0.55 (green color). The landscape was generated by ViaComplex 1.0 software with the following options: plot as "3D-Graph", build on "node", resolution "level-50", contrast "level-50", smoothness "level-50" and zoom "level-50".


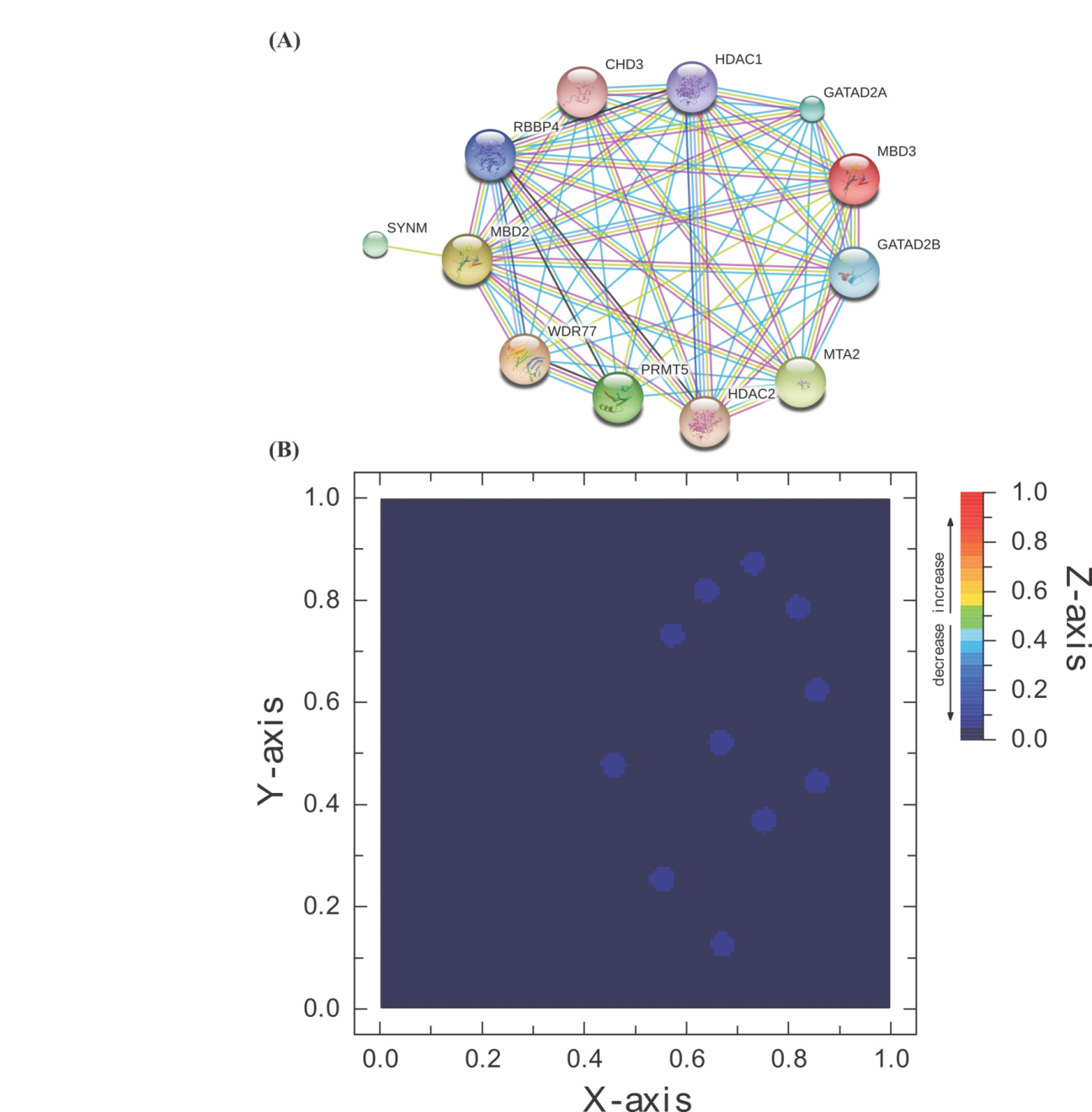


**Figure S6.** Network representation of cluster 7 obtained from STRING metasearch engine (A). This network was used for two-state landscape analysis of gene expression (B). Coordinates (X- and Y-axis) represent normalized values of the input network topology. Color gradient (Z-axis) represents the relative gene functional state mapped onto network according to the transcriptomic data input of GSE30032 series file [placenta plus cord blood transcriptomic data from passive smoker women (a) versus placenta plus cord blood from non-smoker women (b)]. In this sense, the mathematical equation z = a/(a+b) was used to calculated the relative gene functional state of condition (a) and condition (b). Thus, the gene expression in condition (a) is greater than condition (b) when z > 0.55 (yellow to red colors), lower than (b) when z < 0.45 (cyan to blue colors) and equivalent to (b) when 0.45 < z < 0.55 (green color). The landscape was generated by ViaComplex 1.0 software with the following options: plot as "3D-Graph", build on "node", resolution "level-50", contrast "level-50", smoothness "level-50" and zoom "level-50".


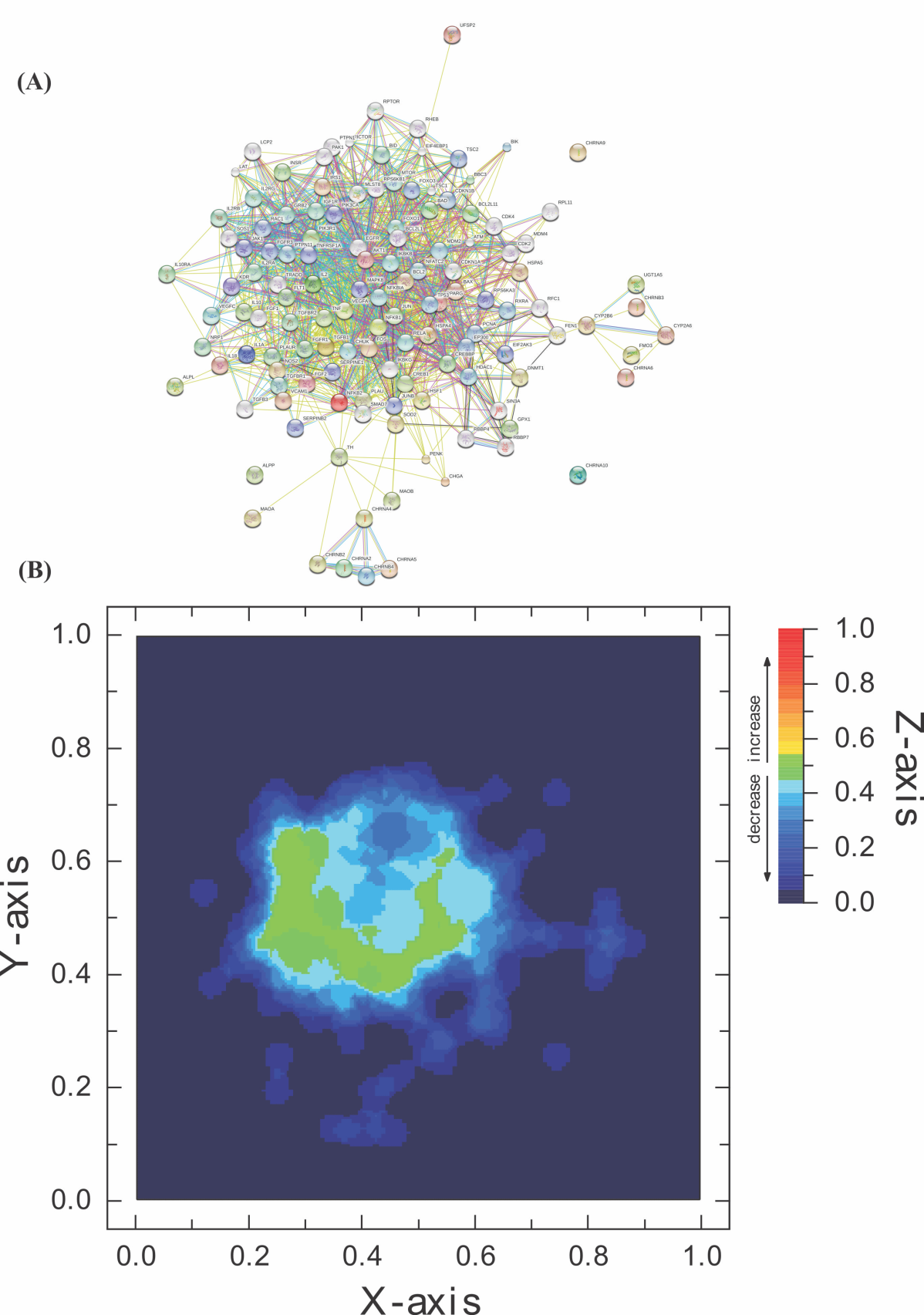


**Figure S7.** Nicotine-associated network obtained from STRING metasearch engine (A). This network was used for two-state landscape analysis of gene expression (B). Coordinates (X- and Y-axis) represent normalized values of the input network topology. Color gradient (Z-axis) represents the relative gene functional state mapped onto network according to the transcriptomic data input of GSE30032 series file [placenta plus cord blood transcriptomic data from passive smoker women (a) versus placenta plus cord blood from non-smoker women (b)]. In this sense, the mathematical equation z = a/(a+b) was used to calculated the relative gene functional state of condition (a) and condition (b). Thus, the gene expression in condition (a) is greater than condition (b) when z > 0.55 (yellow to red colors), lower than (b) when z < 0.45 (cyan to blue colors) and equivalent to (b) when 0.45 < z < 0.55 (green color). The landscape was generated by ViaComplex 1.0 software with the following options: plot as "3D-Graph", build on "node", resolution "level-50", contrast "level-50", smoothness "level-50" and zoom "level-50".


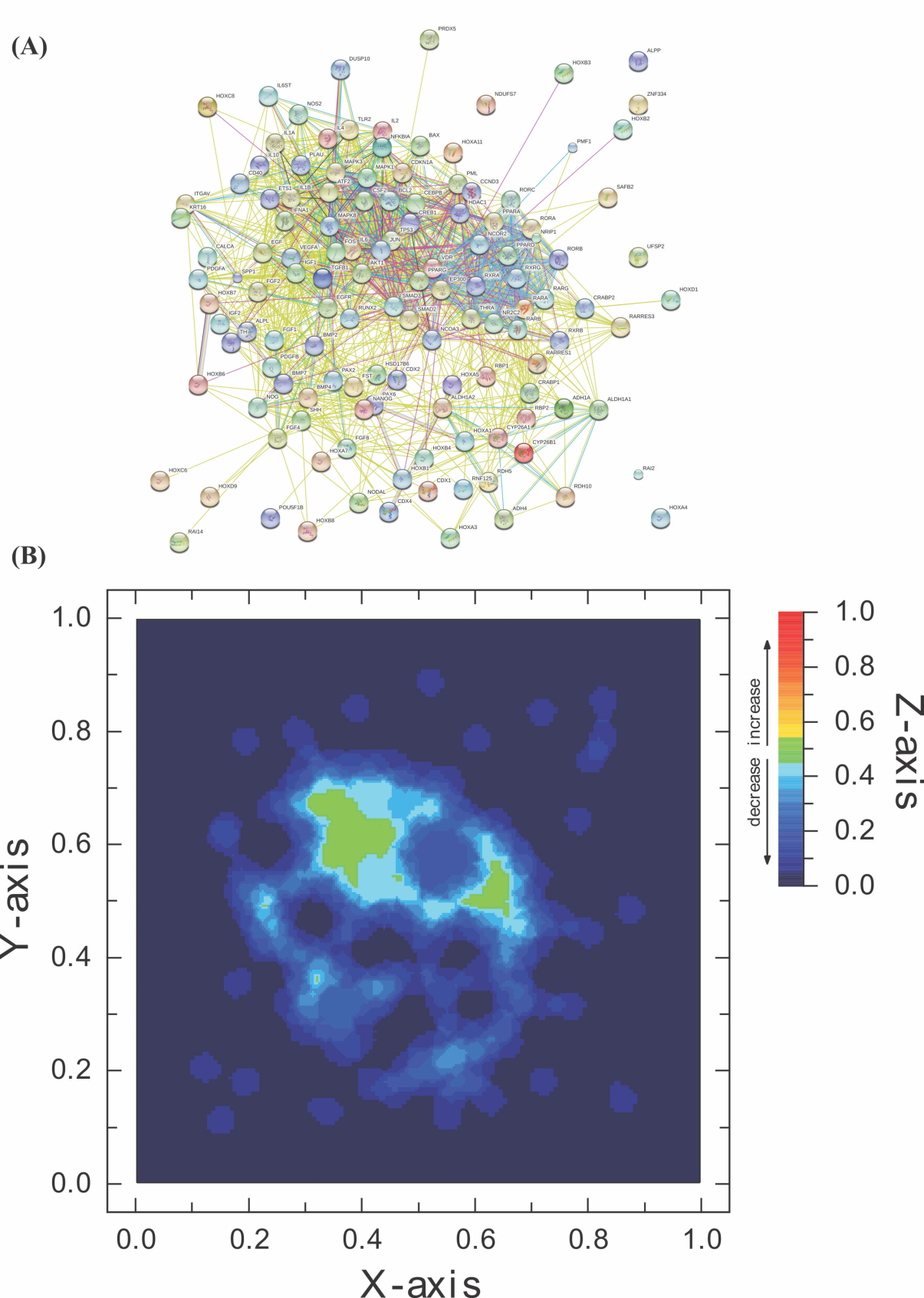


**Figure S8.** Retinoic acid-associated network obtained from STRING metasearch engine (A). This network was used for two-state landscape analysis of gene expression (B). Coordinates (X- and Y-axis) represent normalized values of the input network topology. Color gradient (Z-axis) represents the relative gene functional state mapped onto network according to the transcriptomic data input of GSE30032 series file [placenta plus cord blood transcriptomic data from passive smoker women (a) versus placenta plus cord blood from non-smoker women (b)]. In this sense, the mathematical equation z = a/(a+b) was used to calculated the relative gene functional state of condition (a) and condition (b). Thus, the gene expression in condition (a) is greater than condition (b) when z > 0.55 (yellow to red colors), lower than (b) when z < 0.45 (cyan to blue colors) and equivalent to (b) when 0.45 < z < 0.55 (green color). The landscape was generated by ViaComplex 1.0 software with the following options: plot as "3D-Graph", build on "node", resolution "level-50", contrast "level-50", smoothness "level-50" and zoom "level-50".
